# CTLA4 protects against maladaptive cytotoxicity during the differentiation of effector and follicular CD4^+^ T cells

**DOI:** 10.1038/s41423-023-01027-8

**Published:** 2023-05-09

**Authors:** Yuwei Hao, Bahar Miraghazadeh, Rochna Chand, Ainsley R. Davies, Chelisa Cardinez, Kristy Kwong, Morgan B. Downes, Rebecca A. Sweet, Pablo F. Cañete, Lloyd J. D’Orsogna, David A. Fulcher, Sharon Choo, Desmond Yip, Geoffrey Peters, Sonia Yip, Matthew J. Witney, Maxim Nekrasov, Zhi-Ping Feng, David C. Tscharke, Carola G. Vinuesa, Matthew C. Cook

**Affiliations:** 1 Centre for Personalised Immunology, John Curtin School of Medical Research, The Australian National University, Canberra, ACT Australia; 2 Translational Research Unit, The Canberra Hospital, Canberra, ACT Australia; 3 Division of Immunology and Infectious Diseases, John Curtin School of Medical Research, The Australian National University, Canberra, ACT Australia; 4 Department of Immunology, Fiona Stanley Hospital, Perth, WA Australia; 5 Department of Immunology, The Royal Children’s Hospital, Melbourne, VIC Australia; 6 Department of Medical Oncology, The Canberra Hospital, Canberra, ACT Australia; 7 ANU Medical School, The Australian National University, Canberra, ACT Australia; 8 NHMRC Clinical Trials Unit, The University of Sydney, Sydney, NSW Australia; 9 The ACRF Biomolecular Resource Facility, John Curtin School of Medical Research, The Australian National University, Canberra, ACT Australia; 10 ANU Bioinformatics Consultancy, John Curtin School of Medical Research, The Australian National University, Canberra, ACT Australia; 11 Francis Crick Institute, 1 Midland Rd, London, NW1 1AT UK; 12 Cambridge Institute of Therapeutic Immunology and Infectious Disease, University of Cambridge, Cambridge, United Kingdom

**Keywords:** CTLA4, Immunodeficiency, Cell exhaustion, Terminal differentiation, Cytotoxic CD4^+^ T cells, CD57, Cell death and immune response, Primary immunodeficiency disorders, Follicular T-helper cells

## Abstract

As chronic antigenic stimulation from infection and autoimmunity is a feature of primary antibody deficiency (PAD), analysis of affected patients could yield insights into T-cell differentiation and explain how environmental exposures modify clinical phenotypes conferred by single-gene defects. CD57 marks dysfunctional T cells that have differentiated after antigenic stimulation. Indeed, while circulating CD57^+^ CD4^+^ T cells are normally rare, we found that they are increased in patients with PAD and markedly increased with *CTLA4* haploinsufficiency or blockade. We performed single-cell RNA-seq analysis of matched CD57^+^ CD4^+^ T cells from blood and tonsil samples. Circulating CD57^+^ CD4^+^ T cells (CD4cyt) exhibited a cytotoxic transcriptome similar to that of CD8^+^ effector cells, could kill B cells, and inhibited B-cell responses. CTLA4 restrained the formation of CD4cyt. While CD57 also marked an abundant subset of follicular helper T cells, which is consistent with their antigen-driven differentiation, this subset had a pre-exhaustion transcriptomic signature marked by *TCF7*, *TOX*, and *ID3* expression and constitutive expression of CTLA4 and did not become cytotoxic even after CTLA4 inhibition. Thus, CD57^+^ CD4^+^ T-cell cytotoxicity and exhaustion phenotypes are compartmentalised between blood and germinal centers. CTLA4 is a key modifier of CD4^+^ T-cell cytotoxicity, and the pathological CD4cyt phenotype is accentuated by infection.

## Introduction

Chronic antigenic stimulation is sometimes associated with defective immunity. This has been productively investigated in CD8^+^ T cells, where exhaustion is characterized by the expression of negative regulatory coreceptors, such as CTLA4, PD-1 and Tim-3, and blockade of these receptors with checkpoint inhibitors to restore CD8^+^ T-cell immunity has revolutionised the treatment of some forms of cancer [1–3]. The implications of chronic antigenic stimulation for CD4^+^ T cells are less well characterised. Chronic antigenic stimulation is accentuated in patients with primary antibody deficiency (PAD), which often results in recurrent infections, autoimmunity, or both. This is exemplified by *CTLA4* haploinsufficiency, a Mendelian syndrome of T-cell hyperactivation and autoimmunity that often results in paradoxical and unexplained hypogammaglobulinemia and reduced B cells [4, 5]. We therefore postulated that analysis of T cells from these patients might provide insights into T-cell differentiation that are more difficult to detect under normal circumstances. Furthermore, if PAD accentuates the formation of dysfunctional T cells, this might provide clues regarding the phenotypic diversity that remains unexplained in these patients, even when the underlying genetic cause has been resolved.

T-cell outcomes in response to chronic or recurrent infection include the adoption of dysfunctional phenotypes of senescence, exhaustion and cytotoxicity, which have all been reported to be marked by CD57 expression and/or CD45RA reversion of effector memory cells (T_EMRA_), but the ontogeny, function and significance of these CD4^+^ T-cell outcomes remain to be clearly resolved [6, 7]. This is in contrast with the formation of Th1, Th2 and Th17 effector cells, GC-Tfh cells in secondary lymphoid tissue, and induced Tregs [8, 9], as each subset is well understood at the level of ontogeny, necessary conditions of induction, and transcriptional regulation.

The CD57 epitope arises as a posttranslational modification of various glycoproteins generated by beta-1,3-glucuronyltransferase 1 (GlcAT-P, encoded by *B3GAT1*). Mice express *B3gat1* in the central nervous system but not in lymphocytes. In the human immune system, CD57 was first described as a marker of NK cells [10] and subsequently characterized as a marker of dysfunctional CD8^+^ T cells, which have been reported to exhibit various phenotypic features of exhaustion, cytotoxicity and senescence in both chronic and acute infections [11–14]. CD57^+^ CD4^+^ T cells are normally rare in the blood, although increased proportions have been reported in the context of infections, including COVID-19 [13, 14]. While CD57 is thought to mark CD4^+^ T cells that have undergone antigen-driven expansion, the nature of their phenotype remains controversial, and as with CD57^+^ CD8^+^ T cells, CD57^+^ CD4^+^ T cells have been considered to show various characteristics of exhausted, senescent or cytotoxic phenotypes. In contrast with their rarity in blood, a substantial proportion of germinal center follicular helper T (GC-Tfh) cells are CD57^+^ [15]. Whether CD57 marks a distinct functional subset of GC-Tfh cells is also controversial, and it is also unclear whether CD57 expression marks a state of terminal differentiation in secondary lymphoid organs and how these cells might relate to their circulating counterparts. We previously reported that tonsillar CD57^+^ GC-Tfh cells exhibit a cytotoxic transcriptome that is not evident in CD57^−^ GC-Tfh cells. However, they exhibit a negligible cytotoxic phenotype, with only a very small proportion expressing granzyme proteins [16]. The basis for this discrepancy between transcription and function remains unclear.

Cytotoxic CD4^+^ T cells have been implicated in host defense against virus infection and tumor cells [17, 18] and in the pathology of various diseases, including fibrotic autoimmune and inflammatory diseases [19, 20]. Attempts to resolve the specific characteristics and effector mechanisms of cytotoxic CD4^+^ T cells have revealed variation between humans and mouse models and between infection and cancer contexts. There is evidence to suggest that their formation depends on *RUNX3* and *PRDM1* [21], while other studies have implicated *TBX21* [22]. Expression profiling has indicated that these cells employ granule-mediated cytotoxicity effector mechanisms (*GZMA, GZMB, GZMH, PRF1, FGFB2*), although, in other contexts, *IFNG*, *TNF* and *GNLY* have also been implicated [21, 23, 24]. Conversely, Bcl6 expression may bias against cytotoxicity, as increased numbers of mouse granzyme B^+^ CD4^+^ T cells have been shown to form in response to infection with *Bcl6* deletion, although the population reported did not exhibit expression of other typical cytotoxicity genes (e.g., *Pfr1, Gzma, Gzmm, Gnly, Klg1*) [25].

In summary, CD57^+^ CD4^+^ T cells expand in response to several different types of infection, and CD57 marks cells that have become dysfunctional in response to antigenic stimulation. Thus, we considered this population a candidate for understanding how phenotypes can be modified by chronic antigenic stimulation in patients with PAD and set out to characterize their function and regulation. We found that blood CD57^+^ CD4^+^ T cells were expanded in patients with PAD, particularly in patients with *CTLA4* haploinsufficiency. We found that circulating CD57^+^ CD4^+^ T cells expressed a comprehensive cytotoxic transcriptome similar to that of CD8^+^ T cells. Importantly, these cells can deliver a cytotoxic hit to B cells. CTLA4 appears to be responsible for restraining the emergence of this cell population. Single-cell RNA-seq analysis revealed that in contrast to blood cells, CD57^+^ GC-Tfh cells from tonsils exhibited a transcriptome of precursor exhausted T cells marked by *TCF1*, *TOX* and *ID3* with constitutive high-level expression of CTLA4 and did not become cytotoxic. Thus, CTLA4 mitigates the risk of maladaptive cytotoxicity in the CD4^+^ T-cell compartment.

## Results

### Expansion of CD57^+^ CD4^+^ T cells in human primary antibody deficiency

**Fig. 1:**
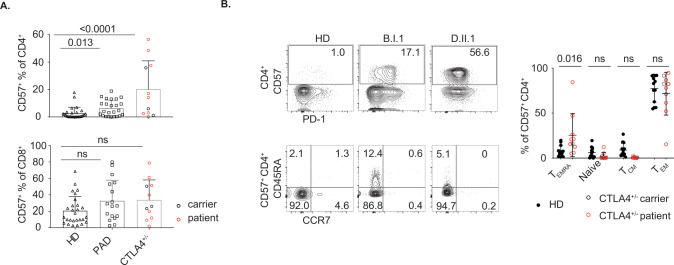
Expansion of CD57^+^ CD4^+^ T cells in samples from patients with *CTLA4* deficiency. **A** Quantification of blood CD57^+^ CD4^+^ and CD57^+^ CD8^+^ T cells derived from PBMCs of healthy donors (*n* = 27), patients with primary antibody deficiency (*n* = 24 for CD4^+^, *n* = 17 for CD8^+^) and *CTLA4*^+/−^ individuals (*n* = 11; carriers - black empty symbols, patients - red empty symbols). **B** Proportions of blood naïve cells (CD45RA^+^ CCR7^+^), T_CM_ (CD45RA^−^ CCR7^+^), T_EM_ (CD45RA^−^ CCR7^−^) and T_EMRA_ (CD45RA^+^ CCR7^−^) in CD57^+^ CD4^+^ T cells from PBMCs of healthy donors (*n* = 13) and *CTLA4*^+/−^ individuals (*n* = 10). Summary data (mean ± SD) were collected from 2 to 3 independent experiments, and statistical analyses were performed by two-tailed unpaired *t*-tests. *P* values are shown. NS not significant

To investigate the effect of chronic antigenic stimulation on T-cell differentiation, we analyzed blood samples from patients with PAD, in which recurrent infections are common and the prevalence of autoimmunity is increased [26]. We observed a significant increase in circulating CD57^+^ CD4^+^ T cells (mean of 5.9% in patients with PAD vs. 2.7% in healthy donors) (Fig. 1A). Furthermore, while there was some patient-to-patient variation, we observed a particularly high proportion of CD57^+^ CD4^+^ T cells (mean of 20.2%) in individuals with heterozygous pathological mutations in *CTLA4* (Fig. 1A). In contrast to CD57^+^ CD4^+^ T cells, there was no significant increase in CD57^+^ CD8^+^ T cells in patients with either PAD or *CTLA4* deficiency (Fig. 1A). Blood CD57^+^ CD4^+^ T cells in *CTLA4*^+/−^ individuals were also predominantly located within CD45RA^−^ CCR7^−^ effector memory (T_EM_) cells subsets, with a small increase in T_EMRA_ cells, which have been reported to exhibit characteristics of senescence, including telomere erosion [27] (Fig. 1B).

*CTLA4*^+/−^ patients present with a syndrome of immune dysregulation, hyperactivation of T cells, and various forms of end-organ pathology. Furthermore, patients with heterozygous mutations in *CTLA4* also often exhibit B-cell defects and hypogammaglobulinemia, and the reason for this remains unclear [4, 5] (Supplementary Fig. 1A and Supplementary Table 1). We confirmed that all *CTLA4* mutations had functional consequences. Nonsense mutations conferred a reduction in CTLA4 expression (Supplementary Fig. 1B, C), while the missense mutation resulted in disrupted ligand-binding capacity with impaired CD80/86 transendocytosis by CTLA4 (Supplementary Fig. 1D).

### Blood CD57^+^ CD4^+^ T cells share a transcriptional signature with cytotoxic CD8^+^ T cells

**Fig. 2:**
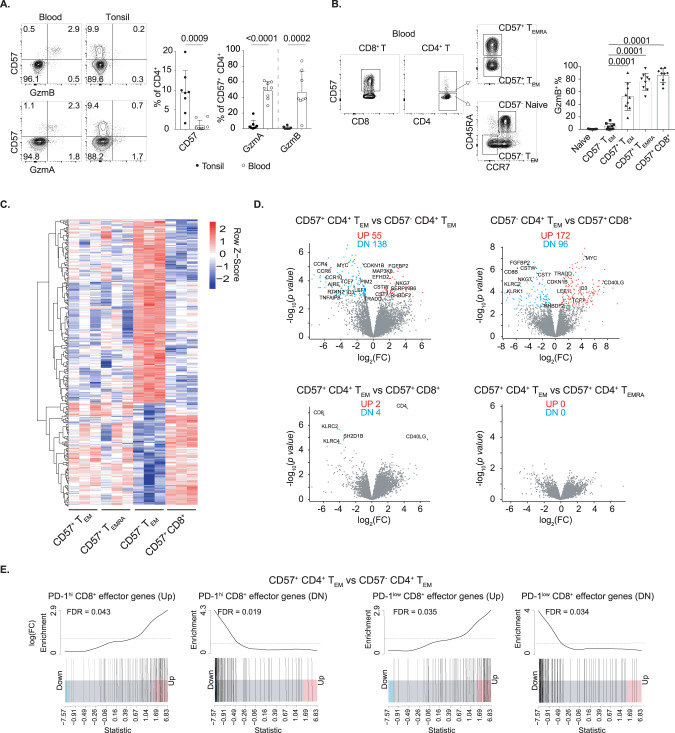
Transcriptome of blood CD57^+^ CD4^+^ cells resembles that of CD57^+^ CD8^+^ cells but differs from that of CD57^−^ CD4^+^ cells. **A** GzmA^+^ and GzmB^+^ cell proportions in CD57^+^ CD4^+^ T cells in tonsil and donor-matched PBMCs (*n* = 8). **B** Flow cytometry gating strategy for CD57^−^ CD4^+^ naive cells, CD57^−^ CD4^+^ T_EM_ (CD45RA^−^ CCR7^−^), CD57^+^ CD4^+^ T_EM_, CD57^+^ CD4^+^ T_EMRA_ (CD45RA^+^ CCR7^−^) and CD57^+^ CD8^+^ T cells and the GzmB expression levels of PBMCs from healthy donors (*n* = 9). **C** Heatmap analysis of RNA sequencing data showing all differentially expressed genes (adjusted *P* values < 0.05) from pairwise comparisons between CD57^−^ CD4^+^ T_EM_, CD57^+^ CD4^+^ T_EM_, CD57^+^ CD4^+^ T_EMRA_ or CD57^+^ CD8^+^ T cells. **D** Volcano plot showing the –log10 (*P* value) and average log-fold change of differentially expressed genes for the indicated comparisons; significant upregulated (red) and downregulated genes (blue) are noted. **E** Gene set enrichment analysis plots for the indicated gene sets (CCR7^−^ PD-1^hi^ or CCR7^−^ PD-1^low^ CD8^+^ cells vs. CCR7^+^ CD45RA^+^ CD8^+^ cells) in the comparison of transcriptomes between CD57^+^ CD4^+^ T_EM_ and CD57^−^ CD4^+^ T_EM_. Data were collected from 2 to 3 independent experiments (mean ± SD). Statistical analyses were performed by two-tailed unpaired *t*-test (**A**) and one-way ANOVA with Bonferroni’s multiple comparison test (**B**). *P* values are shown. NS not significant

CD57 has been inconsistently reported to mark differentiated human CD4^+^ T cells that have adopted exhausted, senescent and/or cytotoxic phenotypes. We aimed to resolve the prevailing uncertainty over the functional significance of CD57^+^ CD4^+^ T cells in the blood and secondary lymphoid organs. We analyzed paired blood and tonsil samples from donors undergoing routine tonsillectomy. A higher proportion of blood CD57^+^ CD4^+^ T cells (~50%) expressed granzyme A and B (GzmA and GzmB), whereas granzyme expression was found in only 1~4% of tonsillar CD57^+^ CD4^+^ T cells (Fig. 2A). High expression of GzmB was observed in the blood CD57^+^ CD4^+^ T_EM_ and T_EMRA_ subsets (Fig. 2B).

To determine whether GzmA and GzmB expression in blood CD57^+^ CD4^+^ T cells reflects activation or is a feature of a broader cytotoxic signature, we purified CD57^+^ CD4^+^ T_EMRA_ and CD57^+^ CD4^+^ T_EM_ from three healthy donors (Fig. 2B) and compared their transcriptomes with those of CD57^−^ CD4^+^ T_EM_ and CD57^+^ CD8^+^ T cells. The transcriptomes of blood CD57^+^ CD4^+^ T_EMRA_ and CD57^+^ CD4^+^ T_EM_ cells were highly similar to those of CD57^+^ CD8^+^ T cells but differed substantially from those of CD57^−^ CD4^+^ T_EM_ (Fig. 2C). A total of 193 genes were differentially expressed (FDR <0.05) between CD57^+^ CD4^+^ T_EM_ and CD57^−^ CD4^+^ T_EM_ subsets, while only 6 genes were differentially expressed between CD57^+^ CD4^+^ T_EM_ and CD57^+^ CD8^+^ cells (Fig. 2D and Supplementary Tables 2–5).

After testing all immunological gene sets from the molecular signatures database (MSigDB) by gene set enrichment analyses (GSEA), we observed significant enrichment of CD8^+^ effector genes in blood CD57^+^ CD4^+^ T cells, indicating that granzyme expression marks a comprehensive cytotoxicity transcriptome in this subset. When compared with CD57^−^ CD4^+^ T_EM_, blood CD57^+^ CD4^+^ T_EMRA_ and T_EM_ were significantly enriched for CD8^+^ T-cell gene signatures, as were both non-naive CCR7^−^ PD-1^hi^ and CCR7^−^ PD-1^lo^ subsets [28] (Fig. 2E and Supplementary Fig. 2A).

Several genes typically expressed in cytotoxic CD8^+^ or NK cells were significantly upregulated in blood CD57^+^ CD4^+^ T_EM_, including *FGFBP2* (also known as *KSP37*) and *CTSW*. We also observed upregulation of *CST7*, *EFHD2* and *NKG7*, which are required for GzmB activity and cytotoxic granule exocytosis by CD8^+^ or NK cells for tumor control in mice [29–31]; *SERPINB6*, which is required for virus clearance in mice because it prevents GzmB-mediated self-injury from cytotoxic granule breakdown [32]; and *RHBDF2* (also known as iRHOM2), which is critical for TNF secretion and activity [33] (Supplementary Fig. 2B). Additional genes potentially involved in the regulation of cell cytotoxicity were also differentially expressed by CD57^+^ CD4^+^ T_EM_ when the criteria were relaxed (FDR <0.1), including *IRF3*, *ARPC2* and *SESN3* (Supplementary Table 3). In contrast, transcripts encoding chemokine receptors *CCR4*, *CCR6*, *CCR8* and *CCR10* and the transcription factor *ID3* were expressed at significantly lower levels in CD57^+^ CD4^+^ T_EM_ (Supplementary Fig. 2B).

### Downregulation of TCF7 in circulating CD57^+^ CD4^+^ T cells

*TCF7* (encoding TCF1) expression distinguishes terminally differentiated exhausted CD8^+^ T cells (T_EX_) and their precursors (precursors of exhaustion, T_PEX_) in the context of chronic infection [34–37]. Deletion of TCF1 in thymic CD4^+^ cells has been shown to reduce ThPOK expression and redirect the cells to a CD8^+^ T-cell fate [38]. We confirmed that when compared to that in CD57^−^ CD4^+^ T cells, TCF1 expression was significantly lower in blood CD57^+^ CD4^+^ T cells from both T_EM_ and T_EMRA_ subsets (Supplementary Fig. 2C). We observed a possible trend toward TCF1 downregulation in blood CD8^+^ T cells, but this was less marked than in CD4^+^ T cells. Together, these data demonstrate that CD57^+^ CD4^+^ T cells marked by TCF1 downregulation exhibit a comprehensive cytotoxic transcriptome that is very similar to that of CD8^+^ effector T cells, and these cells will hereafter be referred to as CD4cyt.

We considered the possibility that the CD4cyt phenotype might arise selectively in Th1 cells. When analyzed based on CXCR3 and CCR6 expression, all four populations were represented. However, the majority of CD57^+^ cells were found to be CXCR3^−^ CCR6^−^ (Supplementary Fig. 2D). Compared with CD57^−^ CXCR3^+^ Th1-like cells, GzmB and perforin expression was restricted to CD57^+^ CD4^+^ T cells with increased T-bet expression (Supplementary Fig. 2E). More importantly, CD27 and CD28 levels were decreased, and KLRG1 levels were increased in CD4cyt, indicating terminal differentiation with reduced proliferative potential [39, 40] (Supplementary Fig. 2E). Analysis of cytokine expression revealed that TNF and IFN-γ were abundant in CD57^+^ CD4^+^ T cells, but IL-4, IL-5, and IL-17A were also detected (Supplementary Fig. 2F). Thus, phenotyping suggested significant enrichment of cytotoxicity-related molecules in CD57^+^ CD4^+^ T cells, but neither chemokine receptor nor cytokine expression showed a clear effector bias.

### CD4cyt cells exhibit limited clonal relatedness to GC-Tfh

In contrast to their rarity in blood, CD57^+^ CD4^+^ T cells are relatively abundant in the tonsil. We investigated whether this subset also exhibited a cytotoxic phenotype. TCF1 has been reported to be expressed by GC-Tfh cells [41, 42], and we confirmed similarly high-level expression in both CD57^+^ and CD57^−^ GC-Tfh (CD45RA^−^ CD25^−^ CXCR5^hi^ PD-1^hi^) and Tfr cells (CD45RA^−^ CD25^+^ CXCR5^hi^ PD-1^hi^). In contrast, analysis of Tfh cells (CD45RA^−^ CD25^−^ CXCR5^int^ PD-1^int^, precursors of GC-Tfh) revealed significantly lower TCF1 expression in CD57^+^ cells than in CD57^−^ cells (Supplementary Fig. 3A, B). Thus, partial TCF1 downregulation is observed in a subset of CXCR5^int^ PD-1^int^ Tfh cells marked by CD57 expression.

**Fig. 3:**
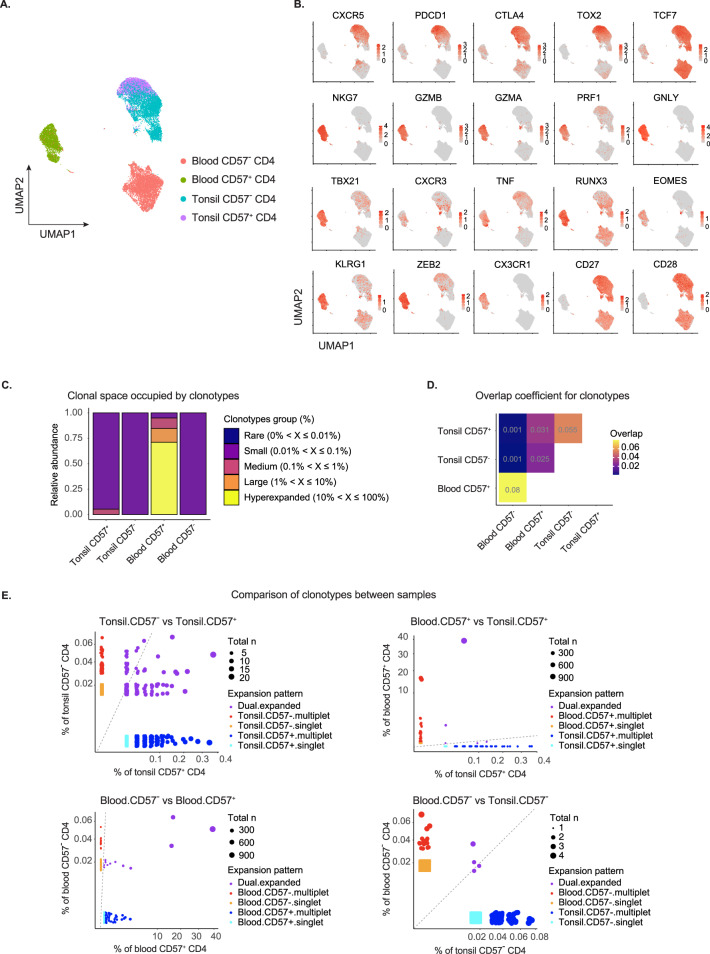
Blood cytotoxic CD57^+^ CD4^+^ T cells are clonally expanded and different from tonsil CD57^+^ GC-Tfh. **A** Uniform manifold approximation and projection (UMAP) plot of the single-cell RNA-seq data from purified CD57^+^ and CD57^−^ CD4^+^ T cells from tonsil (3597 vs. 5056) and donor-matched blood samples (3265 vs. 5953). **B** Relative expression of selected differentially expressed genes related to cytotoxicity and T follicular helper cells. **C** Clonal space homeostasis plot demonstrating the relative space occupied by clonotypes of specific proportions across four different samples (hyperexpanded clonotypes: 10% < X ≤ 100%; large clonotypes: 1% < X ≤ 10%; medium clonotypes: 0.1% < X ≤ 1%; small clonotypes: 0.01% < X ≤ 0.1%; rare clonotypes: 0% < X ≤ 0.01%). **D** Overlap coefficient measurement for the clonotypes across four different samples. **E** Scatter plots comparing the clonotypes between every pair of different samples. The dot sizes correspond to the total number of clones with different colors for the expansion patterns. The relative proportion of clonotypes across all clonotypes is shown with diagonal lines for equal cell fractions

With this suggestion of heterogeneity within the CD57^+^ T-cell compartment of secondary lymphoid organs and blood, we proceeded to perform single-cell RNA-seq analysis of paired blood and tonsillar CD57^+^ and CD57^−^ CD4^+^ T cells. Overall, tonsillar CD57^+^ cells were distinct from CD4cyt and enriched for gene signatures of GC-Tfh (e.g., *CXCR5*, *PDCD1*, *CTLA4, TOX2*, and *TCF7*) (Fig. 3A, B). Cytotoxicity-related transcripts (e.g., *GZMB*, *GZMA*, *NKG7*, *PRF1*, *GNLY*) that were prominently expressed in CD4cyt were not obviously expressed in either tonsillar CD57^+^ or CD57^−^ CD4^+^ T cells (Fig. 3A, B), apart from one very minor subset with limited cytotoxic signatures (e.g., *GZMA*, *NKG7*) (Supplementary Fig. 4A–C), suggesting that the signature detected by the previous RNA-seq analysis [16] was derived from only a few cells.

While blood and tonsillar CD57^+^ cells differentiate down different pathways, we investigated their clonal relatedness. Evaluation of TCR diversity in each compartment revealed that >80% of CD4cyt expressed TCRs from clones that were substantially expanded, while there was no evidence of clonal expansion in CD57^−^ blood cells. In the tonsil samples, there was evidence of limited clonal expansion in CD57^+^ CD4^+^ T cells (Fig. 3C). We observed much greater clonal relatedness between CD57^+^ and CD57^−^ CD4^+^ T cells in tonsil and between CD57^+^ and CD57^−^ CD4^+^ cells in blood, suggesting that precursor-progeny relatedness is relatively common in these subsets (Fig. 3D, E). In contrast, there was limited clonal relatedness between blood and tonsil cells (irrespective of CD57 expression) with few shared clonotypes, although we identified five clones present in both blood and tonsil CD57^+^ CD4^+^ T cells. Thus, under some circumstances, T cells responding to the same antigen can yield progeny that differentiates down both pathways (peripheral effector cells and Tfh) (Fig. 3D, E).

### Single-cell resolution of CD4cyt and CD57^+^ GC-Tfh heterogeneity

**Fig. 4:**
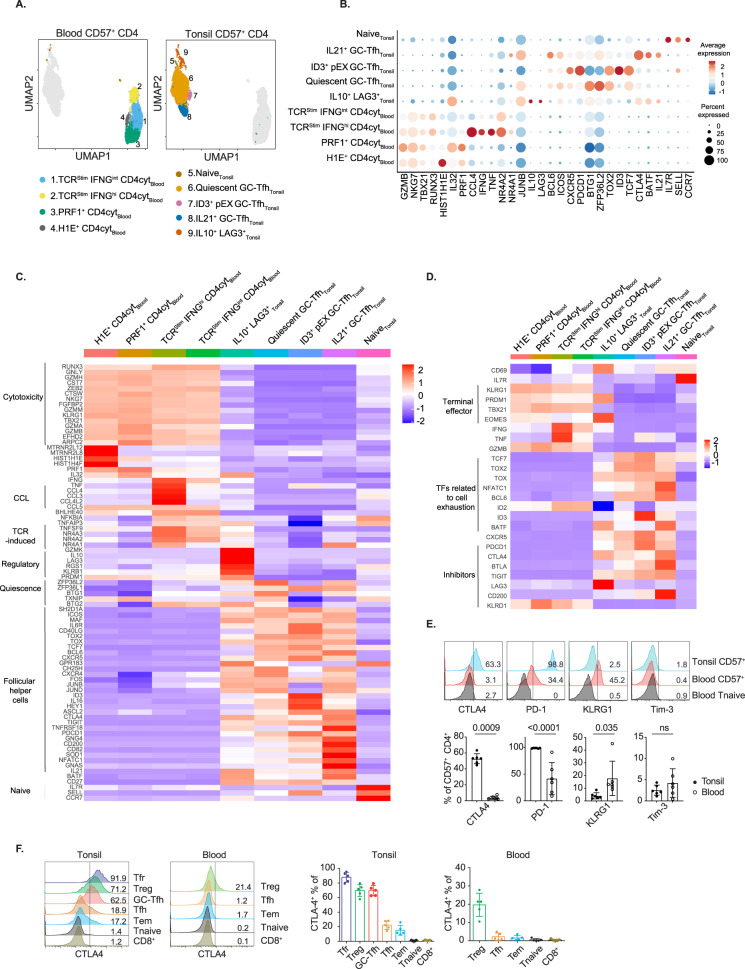
Tonsillar and blood CD57^+^ CD4^+^ T cells are transcriptionally distinct. **A** Uniform manifold approximation and projection (UMAP) plot of the single-cell RNA-seq data from 3597 purified tonsillar and 3265 purified donor-matched blood CD57^+^ CD4^+^ T cells. **B** Dot plot showing markers of the differentially expressed genes between tonsillar and blood CD57^+^ CD4^+^ T cells. **C** Heatmap showing the relative expression of selected markers among the top 200 differentially expressed genes in every blood and tonsillar CD57^+^ CD4^+^ T-cell subset compared to all other subsets. **D** Heatmap showing the relative expression of markers associated with cell exhaustion and terminal differentiation among the top 200 differentially expressed genes. **E** CTLA4, PD-1, KLRG1 and Tim-3 protein expression in CD57^+^ CD4^+^ T cells from tonsil and blood samples (*n* = 6). **F** Flow cytometric analysis of intracellular CTLA4 expression by different T-cell subsets from tonsil and PBMC samples obtained from healthy donors (*n* = 5). The data (mean ± SD) were collected from 2 to 3 independent experiments (**E**, **F**) and generated with two-tailed unpaired *t*-tests. *P* values are shown. NS not significant

Different clusters of CD4cyt were classified according to the expression of *PRF1*, *IFNG* and *NR4A* (Fig. 4A, B), though cytotoxic transcripts were all highly expressed (Fig. 4B, C). Interestingly, the subset expressing high levels of *IFNG* (TCR^Stim^ IFN-γ^hi^ CD4cyt) also highly expressed *NR4A1* (encoding Nur77). Transient expression of NR4A orphan nuclear receptors has been reported in T cells after TCR ligation [43, 44], which could act cell intrinsically to inhibit effector T-cell development and induce hyporesponsiveness [45, 46]. No evidence of cytotoxicity was identified in blood CD57^−^ CD4^+^ T cells, which comprised subsets of naive T cells, CD69^+^ activated effector cells, blood CXCR5^+^ Tfh, and FoxP3^+^ Tregs (Supplementary Fig. 5A–C).

In contrast to CD4cyt, and despite their heterogeneity, there was little evidence of cytotoxicity in tonsil cells. As expected, most tonsillar CD57^+^ CD4^+^ T cells exhibited GC-Tfh signatures [9, 47] (Fig. 4A, B), and they could be further resolved into three subsets: first, classical IL-21^+^ GC-Tfh; second, a subset expressed markers of quiescence (e.g., *ZFP36L1*, *ZFP36L2*, *BTG1*, *BTG2, TXNIP*; markers of reversible cell cycle arrest and basal metabolic rate) [48–50]; and third, a subset marked by high-level expression of *ID3* (Fig. 4B, C). Notably, while CD57^+^ GC-Tfh lacked a cytotoxic signature, they exhibited signatures of cell exhaustion. All tonsillar CD57^+^ GC-Tfh highly expressed inhibitory receptors (e.g., *CTLA4, BTLA, TIGIT, LAG3*) and transcription factors (e.g., *TOX, TOX2, NFATC1, TCF7*), which are critical for the exhaustion of CD8^+^ T cells [1, 34, 51, 52]. High-level *ID3* expression, which is required for the survival and proliferative capacity of CD8^+^ T cells during chronic infection [35, 51], marked the exhausted cell precursors (Fig. 4D). In contrast to tonsil cells, CD4cyt had features of terminal effector cells, characterized by *KLRG1, PRDM1* and *TBX21* expression (Fig. 4D). Thus, blood and tonsil CD57^+^ T cells appear to adopt two separate pathways of CD4^+^ T-cell differentiation and are transcriptionally distinct. Blood CD4cyt are clonally expanded cytotoxic effector cells, while CD57^+^ GC-Tfh have a transcriptome of T_PEX_ and seldom become cytotoxic.

Consistent with the transcriptome data, most tonsillar CD57^+^ CD4^+^ T cells had high protein levels of CTLA4 and PD-1, whereas blood CD4cyt were CTLA4 negative and expressed only a low level of PD-1. We observed KLRG1 expression on blood CD4cyt but not tonsil cells (Fig. 4E). Analysis of CD4^+^ T-cell subsets, however, revealed that CTLA4 was also expressed abundantly on tonsil CD4^+^ T cells. Although CTLA4 is thought to act predominantly by being expressed on Treg cells and CD4^+^ T cells after activation [53], its expression was constitutively abundant on GC-Tfh and Tfr cells, whereas CTLA4 expression was low on their circulating counterparts and negligible on CD8^+^ T cells (Fig. 4F). CTLA4 is a critical negative regulator that inhibits CD80/86 costimulation either by outcompeting CD28 for CD80/86 binding or by inducing removal of CD80/86 via transendocytosis [54, 55]. Consistent with this phenotype, we observed different levels of CD80/86 transendocytosis by tonsillar GC-Tfh, Treg and Tfr subsets, which corresponded to their levels of CTLA4 expression (Supplementary Fig. 6).

### CD4cyt are expanded in patients with *CTLA4* haploinsufficiency

**Fig. 5:**
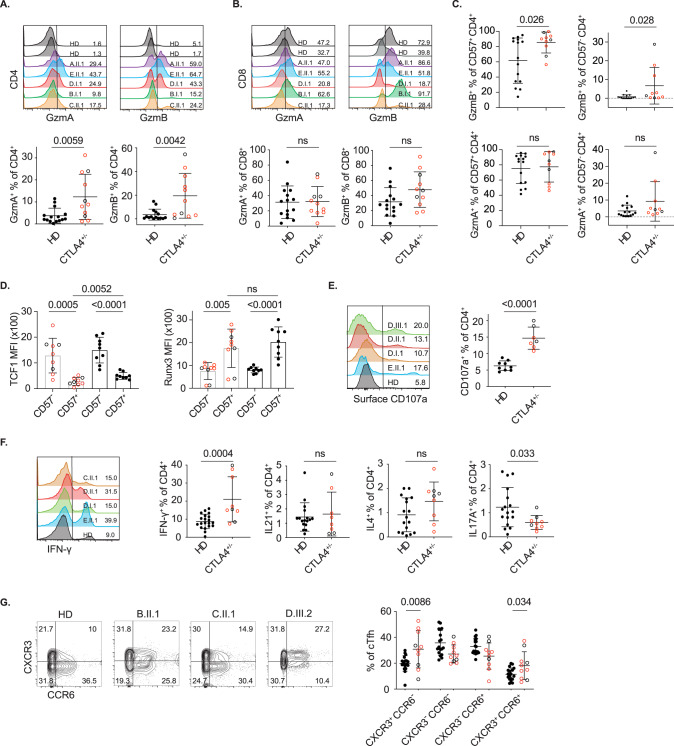
Increased cytotoxicity of CD4^+^ T cells is conferred by human *CTLA4* deficiency. **A**, **B** Representative and summary flow cytometry data of GzmA and GzmB expression in blood CD4^+^ (**A**) and CD8^+^ T cells (**B**) from PBMCs of healthy donors (*n* = 15) and *CTLA4*^+/−^ individuals (*n* = 11). **C** GzmA and GzmB expression in blood CD57^+^ and CD57^−^ CD4^+^ T cells from PBMCs of healthy donors (*n* = 15) and *CTLA4*^+/−^ individuals (*n* = 10). **D** Summary flow cytometry data of TCF1 and Runx3 expression in blood CD57^+^ CD4^+^ and CD57^−^ CD4^+^ T cells from PBMCs of healthy donors (*n* = 9) and *CTLA4*^+/−^ individuals (*n* = 9). **E** Summary flow cytometry data of CD107a expression in blood CD4^+^ T cells from PBMCs of healthy donors (*n* = 9) and *CTLA4*^+/−^ individuals (*n* = 7). **F** Increased proportion of blood CD4^+^ T cells expressing IFN-γ in PBMCs of *CTLA4*^+/−^ individuals (*n* = 9) compared with healthy donors (*n* = 20). **G** CXCR3 and CCR6 expression in blood CXCR5^+^ CD45RA^−^ CD4^+^ Tfh from PBMCs of healthy donors (*n* = 18) and *CTLA4*^+/−^ individuals (*n* = 10). Summary data (mean ± SD) were collected from 2 to 3 independent experiments, and statistical analyses were performed by two-tailed unpaired *t*-tests. *P* values are shown. NS not significant

Having established that CD57 marks bona fide cytotoxic CD4^+^ T cells in the blood, we sought to confirm that the equivalent cells shown to be expanded in *CTLA4*^+/−^ patients exhibited a similar phenotype. We demonstrated a substantial expansion of GzmA^+^ and GzmB^+^ CD4^+^ T cells in *CTLA4*^+/−^ individuals (Fig. 5A), while the abundance of GzmA^+^ and GzmB^+^ CD8^+^ T cells was not different from that in healthy donors (Fig. 5B). Almost all CD4cyt from *CTLA4*^+/−^ individuals were GzmB^+^, and this was more marked in *CTLA4*^+/−^ donors than controls, whereas most CD57^−^ cells were Gzmb^−^ (Fig. 5C). CD4cyt from *CTLA4*^+/−^ individuals exhibited lower TCF1 expression and increased Runx3 expression than CD57^−^ CD4^+^ T cells (Fig. 5D). CD4cyt from *CTLA4*^+/−^ donors also exhibited increased expression of CD107a, a marker of cytotoxic degranulation (Fig. 5E). We also observed an increased proportion of IFN-γ-producing cells and a reduction in IL-17A^+^ cells, while the proportions of IL-4- and IL-21-producing cells were not different from those in healthy donors (Fig. 5F). The percentage of CXCR3^+^ cells in blood CXCR5^+^ Tfh was also significantly increased (Fig. 5G). Taken together, these data demonstrate the expansion of CD4cyt and increased cytotoxic features in blood CD4^+^ T cells, which are previously unappreciated features of patients with *CTLA4* haploinsufficiency.

### CTLA4 inhibits the acquisition of cytotoxic features by CD4^+^ T cells

**Fig. 6:**
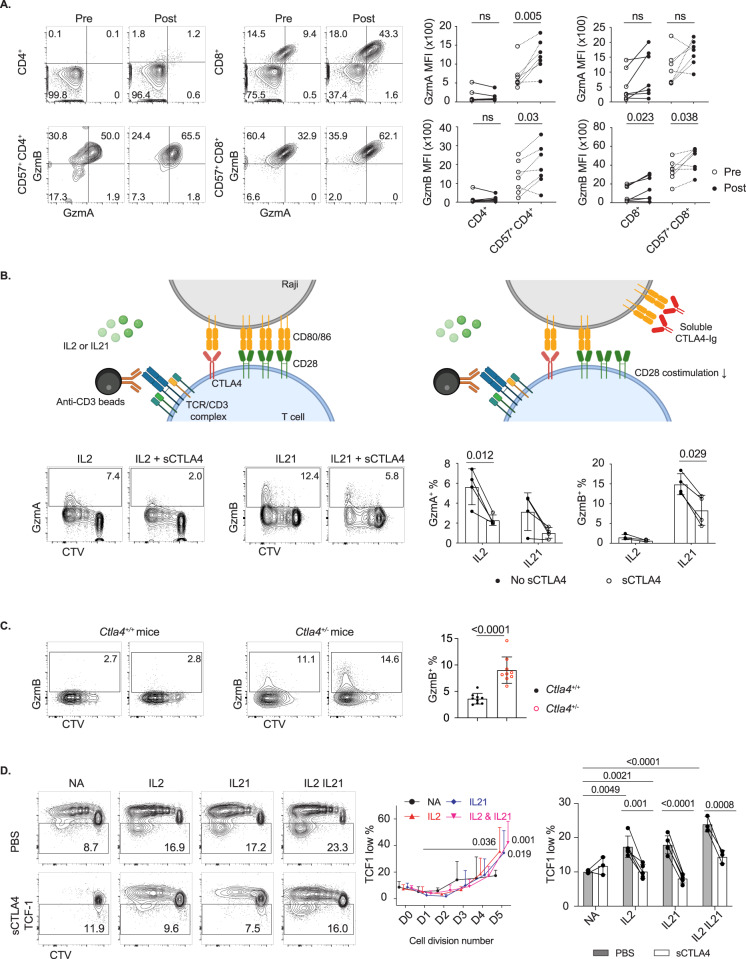
CTLA4 inhibits the acquisition of cytotoxic features by CD4^+^ T cells. **A** Increased GzmA and GzmB expression in CD57^+^ CD4^+^ T cells from PBMCs of cancer patients (*n* = 7) after treatment with ipilimumab/nivolumab (anti-CTLA4/PD-1). Each symbol represents one cancer patient before (empty symbols) and after (filled symbols) treatment. **B**, **D** Tonsillar CD4^+^ T_EM_ were stimulated with anti-CD3 beads, CD80/86-expressing irradiated Raji cells and IL-2 or IL-21 for 4 days. **B** Suppression of induced GzmA and GzmB expression in tonsillar CD4^+^ T_EM_ cells by soluble CTLA4-Ig (*n* = 4). CTLA4-Ig binds to CD80/86 with higher affinity than CD28 to suppress costimulation (as shown in the diagram). **C** Increased GzmB expression in CD4^+^ T cells from *Ctla4*^+/−^ mice. *Ctla4*^+/+^ or *Ctla4*^+/−^ CD4^+^ T cells were stimulated with plate-coated anti-CD3, B cells (4T: 1B), IL-2 and IL-21 for 5 days. **D** Suppression of induced TCF1^low^ cell formation from tonsillar CD4^+^ T_EM_ by soluble CTLA4-Ig (*n* = 3–4). Summary data (mean ± SD) were collected from 2 to 3 independent experiments, and statistical analyses were performed by two-tailed unpaired *t*-tests (**B** and **C**), two-tailed paired *t*-tests (**A**) and two-way ANOVA with Bonferroni’s multiple comparison tests (**D**). *P* values are shown. NS not significant

Next, we investigated whether differential expression of the inhibitory receptors CTLA4 and PD-1 might account for different levels of cytotoxic features between blood and tonsillar CD57^+^ CD4^+^ T cells. Immune checkpoint inhibitors (ICIs) act specifically to block CTLA4 and PD-1 [56, 57], so we compared GzmA and GzmB expression in CD57^+^ CD4^+^ T cells in blood obtained from cancer patients before and after treatment with anti-CTLA4/PD-1 (ipilimumab/nivolumab) combination checkpoint inhibitor therapy. We identified that GzmA and GzmB expression levels in blood CD4cyt cells were increased after CTLA4 and PD-1 blockade (Fig. 6A), while the proportion showed no significant changes (Supplementary Fig. 7A). An increase in GzmB expression was also observed in the CD8^+^ T-cell compartment, but there was no change in GzmA expression in CD8^+^ T cells after treatment (Fig. 6A).

The increased granzyme expression could have been the result of blocking either CTLA4 or PD-1 or both by ICIs, so we specifically examined the action of CTLA4 inhibition in vitro. Soluble CTLA4-immunoglobulin (CTLA4-Ig) was administered to bind to CD80/86 with higher affinity than CD28, preventing CD28-mediated costimulation [58]. CD4^+^ T_EM_ were stimulated with anti-CD3 antibody, CD80/86-expressing irradiated Raji cells and different cytokines. IL-2 and IL-21 were identified as the most potent inducers of GzmA and GzmB, respectively (Supplementary Fig. 7B). The addition of soluble CTLA4-Ig significantly reduced the induction of GzmA and GzmB expression (Fig. 6B). We also performed an experiment comparing the responses of CD4^+^ T cells collected from *Ctla4* haploinsufficient mice and their WT counterparts, and the results confirmed that CTLA4 restricted GzmB induction (Fig. 6C).

We examined whether the same culture conditions also resulted in a change in TCF1 expression. Stimulation of CD4^+^ T_EM_ with IL-2 and IL-21 alone or in combination resulted in a proliferation-dependent population of TCF1^low^ cells. This phenotype was also influenced by CTLA4, and the emergence of TCF1^low^ CD4^+^ T cells was reduced after the addition of soluble CTLA4-Ig (Fig. 6D). In contrast, tonsillar CD57^+^ CD4^+^ T cells exhibited defective proliferation and were resistant to upregulation of GzmA and GzmB upon stimulation, consistent with other evidence suggesting cell exhaustion (Supplementary Fig. 7C).

### CTLA4 blockade increases CD4^+^ T-cell cytotoxicity in VACV-infected mice

**Fig. 7:**
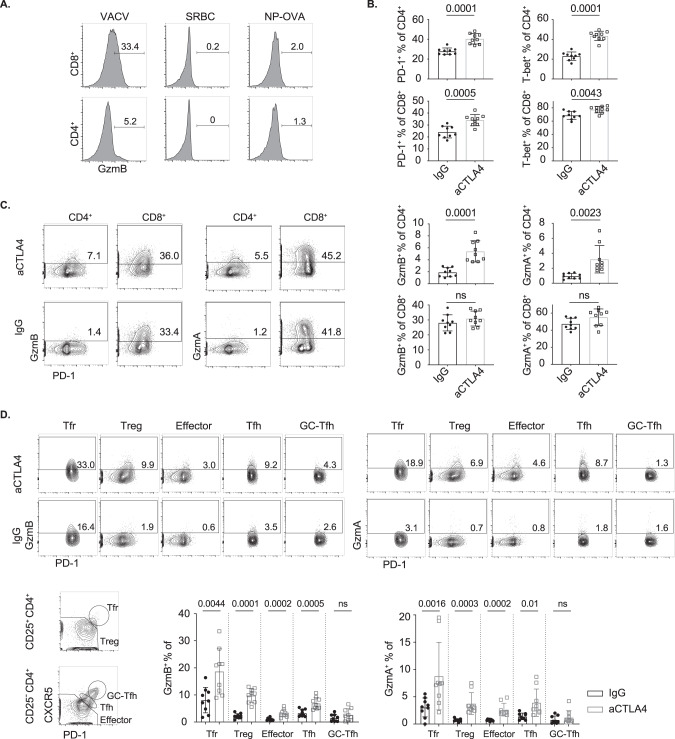
Increased granzyme expression after CTLA4 blockade in VACV-infected mice. **A** GzmB expression in T cells 7 days postimmunization. C57BL/6 mice were i.v. injected with 2 × 10^8^ sheep red blood cells, i.p. injected with 100 μg NP-OVA (1:1 with alum) or i.p. injected with 1 × 10^6^ pfu VACV. **B**–**D** C57BL/6 mice were i.p. injected with 1 × 10^6^ pfu VACV at Day 0, 100 μg anti-mouse CTLA4-blocking antibody or isotype IgG was injected into mice at Days –1, 2 and 5, and spleens were collected at Day 7. **B** Increased PD-1 and T-bet expression by splenic CD4^+^ and CD8^+^ T cells (*n* = 9) from mice treated with anti-CTLA4 antibody compared with IgG. **C** Increased GzmA and GzmB expression by splenic CD4^+^ but not CD8^+^ T cells (*n* = 9) from mice treated with anti-CTLA4 antibody compared with IgG. **D** GzmA and GzmB expression in splenic live Tfr (CD4^+^ CD8^−^ CD25^+^ CXCR5^hi^ PD-1^hi^), Treg (CD4^+^ CD8^−^ CD25^+^ CXCR5^int/low^ PD-1^int/low^), GC-Tfh (CD4^+^ CD8^−^ CD25^−^ CXCR5^hi^ PD-1^hi^), Tfh (CD4^+^ CD8^−^ CD25^−^ CXCR5^int^ PD-1^int^) and effector cells (CD4^+^ CD8^−^ CD25^−^ CXCR5^low^ PD-1^low^) (*n* = 9) from mice treated with anti-CTLA4 antibody compared to IgG. Summary data (mean ± SD) were collected from 3 independent experiments, and statistical analyses were performed by two-tailed unpaired *t*-tests. *P* values are shown. NS not significant

Next, we investigated the effect of CTLA4 antagonism on CD4 cytotoxicity with a model of acute infection. First, we determined the conditions under which cytotoxic CD4^+^ T cells could be induced. GzmB is usually detectable in a subset of human CD8^+^ T cells. However, GzmB was not detectable on splenic CD8^+^ T cells isolated from mice housed in specific pathogen-free (SPF) conditions. Furthermore, immunization with the model antigen NP-OVA or sheep red blood cells failed to upregulate GzmB in either CD8^+^ or CD4^+^ T cells, whereas infection with the vaccinia virus Western Reserve strain (VACV) induced GzmB expression in a subset of both CD4^+^ and CD8^+^ T cells (Fig. 7A).

To investigate whether CTLA4 inhibits the emergence of CD4^+^ T-cell cytotoxicity in vivo, an anti-CTLA-4 antibody, which blocks CTLA4 and therefore promotes CD28 costimulation, was applied. Mice were infected with VACV and treated with anti-CTLA4 antibody or isotype control IgG before and after infection. We observed increased expression of PD-1 and T-bet in T cells in the presence of anti-CTLA4 blockade (the expression of CD57 on mouse lymphocytes was not characterised) (Fig. 7B). Compared with mice receiving the isotype control, mice treated with anti-CTLA4 antibody showed significantly increased expression of both GzmA and GzmB. Interestingly, this was not observed in CD8^+^ T cells (Fig. 7C). Furthermore, Tfr, Treg, Tfh and T_EM_ exhibited increased GzmA and GzmB expression after anti-CTLA4 treatment. However, similar changes were not observed in GC-Tfh, suggesting that they remained relatively refractory to the adoption of the cytotoxic phenotype (Fig. 7D). In summary, infection but not model antigens was able to induce CD4^+^ T-cell cytotoxicity in vivo, and this phenotype was accentuated by administration of anti-CTLA4 antibody.

### CD4cyt mediate B-cell death

**Fig. 8:**
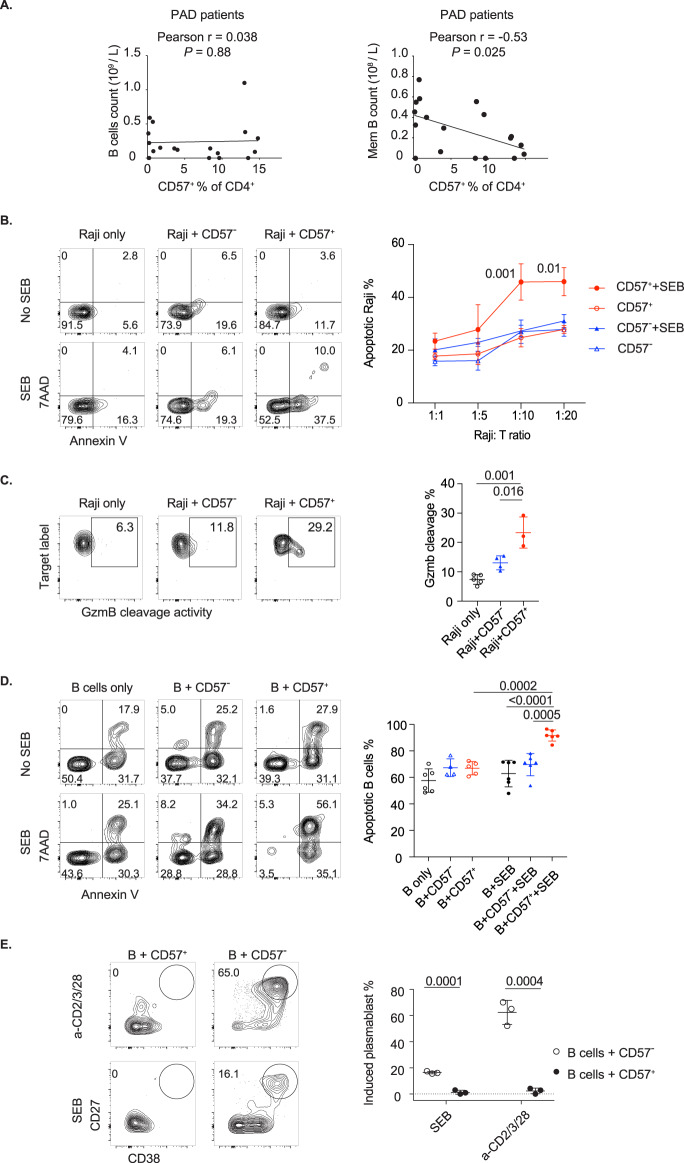
Cytotoxic blood CD57^+^ CD4^+^ T cells kill B cells. **A** Analyses of the correlation of blood B-cell and memory B-cell counts with the proportions of blood CD57^+^ CD4^+^ cells in patients with PAD (*n* = 18). **B**–**E** CD57^+^ CD4^+^ and CD57^−^ CD4^+^ T cells were purified from PBMCs of healthy donors. **B** Increased Raji cell apoptosis was mediated by blood CD57^+^ CD4^+^ T cells. Raji cells were pulsed with SEB or not and cocultured with CD57^+^ CD4^+^ or CD57^−^ CD4^+^ T cells (*n* = 4–6) at the indicated cell ratios for 24 h. **C** GzmB activity in Raji cells incubated with blood CD57^+^ CD4^+^ T cells. SEB-pulsed Raji cells were cocultured with CD57^+^ CD4^+^ or CD57^−^ CD4^+^ T cells (10 T:1 Raji; *n* = 3–5) and GzmB fluorogenic substrate for 2 h. **D** Increased B-cell apoptosis was mediated by CD57^+^ CD4^+^ T cells. Blood B cells were stimulated with SEB or not and cocultured with CD57^+^ CD4^+^ or CD57^−^ CD4^+^ T cells (10 T:1 B; *n* = 4–6) for 24 h in the presence of IL-4 and IL-21. **E** Reduced plasmablast formation from B cells incubated with blood CD57^+^ CD4^+^ T cells. Blood B cells were stimulated with SEB or anti-CD2/3/28 beads and cocultured with CD57^+^ CD4^+^ or CD57^−^ CD4^+^ T cells (1T: 1 B; *n* = 3) for 6 days in the presence of IL-4 and IL-21. Summary data (mean ± SD) were collected from 2 to 3 independent experiments, and statistical analyses were performed by two-tailed unpaired *t*-tests (**C**, **E**), two-way ANOVA with Bonferroni’s multiple comparison tests (**B**, **D**) and Pearson correlation coefficient analyses (**A**). *P* values are shown. NS not significant

*CTLA4*^+/−^ patients exhibit defects in B cells and hypogammaglobulinemia, and the reason for these characteristics remains unclear [4, 5]. CD4^+^ T cells with upregulated cytotoxic features have been shown to mediate MHC class II-dependent elimination of virus-infected cells and tumor cells [24, 59], suggesting that B cells may kill via cognate interactions with CD4cyt. We observed an inverse relationship between CD4cyt and memory B-cell counts in PAD patients (Fig. 8A). Furthermore, CD4cyt were highly expanded in blood samples from *CTLA4*^+/−^ patients, in whom hypogammaglobulinemia and deficiencies of total B cells or B-cell subsets were common (Supplementary Table 6). Despite the described B-cell deficiencies, *CTLA4*^+/−^ individuals had increased CD4^+^ T-cell activation with reduced naive CD4^+^ T cells and increased PD-1 expression, decreased circulating CD25^+^ Foxp3^+^ Treg cells and unchanged levels of TNF-producing CD4^+^ and CD8^+^ T cells (Supplementary Fig. 8A–D).

To test whether CD4cyt mediate B-cell killing through cognate interactions, we cocultured Raji cells (a human B lymphoblastoid cell line) with CD4cyt from healthy donors and assessed cell apoptosis with annexin V staining. Raji cells were pulsed with staphylococcal enterotoxin B (SEB), which facilitated immune synapse formation between Raji cells and CD4^+^ T cells [60]. We observed a dose-dependent increase in the apoptosis of SEB-pulsed Raji cells when they were cocultured with blood CD57^+^ CD4^+^ T cells compared to when they were cocultured with noncytotoxic CD57^−^ CD4^+^ T cells or T cells from healthy donors (Fig. 8B). A minimal increase in apoptosis of Raji cells was observed without SEB treatment, suggesting that the cognate interaction between CD4cyt and target cells is necessary for cytotoxic activity. By applying a cell-permeable and granzyme B-specific fluorogenic substrate [61], we detected GzmB activity in SEB-pulsed Raji cells incubated with blood CD4cyt, and the results indicated that they could kill target cells via GzmB (Fig. 8C). A similar effect was observed with primary B cells. We observed a significant increase in the apoptosis of SEB-stimulated B cells when they were incubated with autologous blood CD4cyt but no cytotoxic activity of CD57^−^ CD4^+^ T cells (Fig. 8D). Finally, CD4cyt did not aid B-cell differentiation in vitro. Fewer plasmablasts were formed from B cells incubated with autologous CD4cyt in the presence of SEB or anti-CD2/3/28 antibody with IL-4 and IL-21 (Fig. 8E). Together, these data reveal that CD4cyt are unable to aid B cells, and indeed, they deliver a cytotoxic hit to them.

## Discussion

We have identified remarkable similarities between the transcriptomes and protein expression profiles of CD4cyt and CD8^+^ effector cells related to cytotoxic function. Several lines of evidence indicate that CTLA4 is instrumental in preventing cytotoxic CD4^+^ T-cell formation. CD4cyt are expanded in patients with heterozygous *CTLA4* mutations, and the cytotoxicity of CD4^+^ T cells is increased in cancer patients after ICI therapy and in a mouse model of acute viral infection after CTLA4 blockade. Interestingly, this effect is relatively specific to CD4^+^ T cells. CTLA4 expression by CD8^+^ T cells is negligible in human blood and tonsils, while it is induced after activation of blood CD4^+^ T cells, and CTLA4 is constitutively expressed at high levels by all nonnaive CD4^+^ T-cell subsets (Tfr, Tfh, Treg) in tonsils. Consistent with this, we failed to identify increased cytotoxicity of CD8^+^ T cells in patients with *CTLA4* haploinsufficiency or after CTLA4 blockade in VACV-infected mice.

Acquisition of an effector phenotype almost equivalent to that of CD8^+^ T cells means that the cytotoxic effector program is accessible to CD4^+^ T cells; however, based on the relative abundance of CD4cyt, the threshold for adopting this phenotype appears to be higher for CD4^+^ T cells than for their CD8^+^ counterparts. GC-Tfh appear to have an even higher threshold for becoming cytotoxic. We showed that after live viral infection of mice, GC-Tfh were particularly biased against the emergence of cytotoxic features. There are, however, circumstances under which cytotoxic Tfh can form. For example, they have been found in mice after the deletion of regulatory cells [62] and in children with recurrent group A *Streptococcus* tonsillitis [63]. Nevertheless, even though they emerge after chronic antigenic stimulation, as indicated by CD57 expression and expression of *NR4A1*, which provides evidence of recent T-cell receptor ligation [43, 44], very few tonsillar CD57^+^ GC-Tfh have a cytotoxic transcriptome.

GC-Tfh constitutively express CTLA4, which is part of the transcriptional signature for precursors of exhaustion. Several pathways might account for the acquisition of this signature with no cytotoxicity in GC-Tfh. First, IL-2 is a negative regulator of GC-Tfh formation, whereas we showed that CD4cyt are formed in a proliferation-dependent manner in response to IL-2. IL-2 promotes cytotoxicity in both CD4^+^ and CD8^+^ T cells, and in CD8^+^ T cells, this occurs via induction of *Eomes* and *Prdm1* expression [64–66]. Furthermore, in the tumor microenvironment, Treg-dependent regulation of IL-2 was demonstrated to be critical for the acquisition of cytotoxic features by tumor-infiltrating CD4^+^ T cells [67]. In contrast, loss of IL-2 promotes exhaustion [68]. Second, Bcl6 promotes the generation of memory CD8^+^ T cells and GC-Tfh differentiation in CD4^+^ cells and inhibits CD8^+^ effector cell formation and granzyme expression by CD4^+^ and CD8^+^ T cells [25, 69–73].

TCF1 expression also appears to be crucial for the differences between CD4cyt and GC-Tfh. GC-Tfh appear to be refractory to stimuli that downregulate TCF1 in peripheral CD4^+^ T cells. Comparison of T-cell subsets from blood and tonsils revealed a reduction in TCF1 expression in all CD57^+^ CD4^+^ T-cell subsets but not in GC-Tfh. High *Tcf7* expression is important for GC-Tfh formation [41, 42], while high expression of BCL6, which inhibits non-Tfh differentiation, antagonizes *Tcf7* downregulation via repression of *Klf2* [47]. In contrast, we noted significantly reduced transcript and protein expression of TCF1 in CD4cyt_._ This trend was more obvious in patients with *CTLA4* deficiency. Conversely, CTLA4 prevents TCF1 downregulation and the acquisition of cytotoxicity during CD4^+^ effector cell activation. In CD8^+^ T cells, high TCF1 expression is also associated with stemness and lower levels of inhibitory receptor (e.g., PD-1, Tim-3) expression [74]. In mice, deletion of *Tcf1* and *Lef1* leads to increased transcript levels of the cytotoxic molecules *Gzmb*, *Gzmk* and *Prf1* in CD8^+^ T cells and Tregs [38, 75, 76]. TCF1 downregulation has also been observed in terminally differentiated effector Tregs, which are characterized by a transcriptional program similar to that identified for CD4cyt in this study, with increased *Gzmb* and *Tbx21* transcript levels [77], indicating a potential causal relationship between loss of TCF1 and increased cytotoxicity of T cells.

While GC-Tfh are refractory to becoming cytotoxic, features of cytotoxicity can be observed in Th1 and Th2 effector CD4^+^ T-cell subsets, although these effectors need to be sufficiently abundant to permit the identification of a small subset of terminally differentiated cells. For example, spontaneously expanded GATA-3^+^ Th2 in *Foxp3*^null^ mice express high levels of granzyme B [78]. Th1 can also become cytotoxic, but together, these findings suggest that not all cytotoxic CD4^+^ T cells form downstream of Th1 [17, 25, 79]. Our findings do not exclude the possibility of a precursor-progeny relation with Th1 cells, but we identified substantial differences between Th1 and CD4cyt. Thus, while some CD4cyt express *TBX21*, their comprehensive cytotoxic transcriptome does not overlap significantly with the Th1 transcriptome. Furthermore, only a minority (~10%) of CD4cyt are CXCR3^+^, while a greater proportion are CXCR3^−^ CCR6^−^. This expression pattern parallels that in the CD8^+^ T-cell compartment, where most antigen-specific cells express *Ifng*, but this does not correlate with *Prf1* expression or cytolytic activity [80].

Cytotoxic CD4^+^ T cells have been shown to kill ectromelia virus-infected targets based on peptide/MHC class II^+^ recognition and perforin-dependent cytotoxicity [59]. Similarly, intratumoral cytotoxic CD4^+^ T cells have been shown to mediate MHC class II-dependent killing of human bladder cancer cells [24]. We showed in vitro that unlike other CD4^+^ T-cell subsets, CD4cyt inhibit B-cell responses and induce B-cell death. Consistent with these findings, we showed that in humans, CD4cyt are more abundant in patients with chronic antigenic stimulation (PAD) than in healthy controls. We identified an inverse relationship between memory cells and CD4cyt in the context of immune deficiency. We speculate that the aberrant action of CD4cyt can occur instead of T-cell help during the activation of memory B cells, resulting in their loss and preventing the formation of plasmablasts, but this will require further investigation.

CD4^+^ T-cell cytotoxicity appears to be promoted by primary immune deficiency, particularly *CTLA4* deficiency and antagonism. Furthermore, we found an inverse relationship between CD4cyt and memory B cells in patients with PAD. These findings raise the possibility that CD4cyt could contribute to the otherwise unexplained immune deficiency that emerges in *CTLA4* haploinsufficiency and in other immune dysregulation syndromes where autoimmunity occurs concurrently with antibody deficiency, for example, syndromes related to *STAT3* gain-of-function [4, 5, 81]. An understanding of CD4cyt formation could also provide new insights into immune pathology arising from gene‒environment interactions. In addition, the development of CD4cyt might contribute to the immune paralysis seen with chronic infection in patients who are apparently immune competent. Indeed, COVID-19 patients infected by SARS-CoV-2 have been reported to have increased percentages of CD57^+^ PD-1^+^ CD4^+^ cells, which might increase throughout the infection [13, 14], and disrupted germinal center reactions with decreased BCL6-expressing Tfh and GC B cells and reduced total CD3^+^ T- and B-cell counts have also been reported in acute COVID-19 patients [82].

Finally, our findings support the idea that the risk of maladaptive immunity can be robust to perturbation by single genetic variants but emerge in the context of chronic antigenic stimulation. The formation of CD4cyt is normally constrained, as these cells are rare in blood from healthy donors but expanded in patients with PAD, even more so in the subset of cells generated by *CTLA4* haploinsufficiency. Furthermore, in mice, cytotoxic CD4^+^ T cells normally only arise after strong antigenic stimulation but not after immunization with model antigens. Together, these observations illustrate how the emergence of cellular and even disease phenotypes can result from a sustained antigenic challenge when genetic variation confers a potential breach in immune regulation.

## Materials and methods

### Human blood and tonsil samples

Healthy adults without a family history of immune diseases were recruited for blood collection, and PBMCs were collected by gradient centrifugation (15 min, 2000 rpm and no brakes) using Ficoll-Paque Plus tubes (GE Healthcare). Children with routine tonsillectomy were recruited for tonsil collection, and their blood may also have been obtained. After mechanical disruption by surgical blades, tonsillar lymphocytes were also collected by gradient centrifugation (30 min, 400 g and no brakes). Patients with recurrent infections and diagnosed with PAD were recruited through the Australia and New Zealand Antibody Deficiency Allele study. Patients with *CTLA4* mutations were recruited through the Centre for Personalised Immunology at ANU, which studies immune diseases by discovering causative genetic variation. The patients and healthy donors were matched for age and sex. All human samples were collected with informed consent, and the experimental procedures were approved by the Australian National University Human Experimentation Ethics Committee and the Australian Capital Territory Health Human Research Ethics Committee.

### Patients receiving combination checkpoint inhibitor therapy

Blood samples from six melanoma and one lung cancer patient with stage IV disease were collected before and 6–10 weeks after the treatment. Nivolumab was normally given at 1 mg/kg, and ipilimumab was given at 3 mg/kg on a 3-week cycle for 2–4 cycles, except for the patient with lung cancer, who received nivolumab 3 mg/kg every 2 weeks and ipilimumab 1 mg/kg every 6 weeks. The study was approved by the Australian National University Human Experimentation Ethics Committee and the Australian Capital Territory Health Human Research Ethics Committee.

### Mice

C57BL/6 mice were bred and maintained in the Australian Phenomics Facility at ANU under SPF conditions. Female mice aged 8–10 weeks were utilised for VACV infection and anti-CTLA4-immunoglobulin injection. Mouse splenocytes were collected in cold RPMI-1640 (Thermo Fisher) with 10% FBS (Thermo Fisher) after gentle disruption of the spleen by syringe plunger and passage through 70 μm filters (Miltenyi Biotec), followed by red blood cell lysis using lysing buffer (BD).

*Ctla4* knockout mice were generated from C57BL/6 mice by CRISPR technology according to a previously published protocol [83]. Two single-guide RNAs (sgRNAs) (guide 1: 5’-AGAAGTCCTCTTACAACAGGGG-3’, guide 2: 5’-GTACCCACCGCCATACTTTGTGG-3’) were designed by CCTop and CRISPOR and created a 1926-bp deletion in *Ctla4* between exons 2 and 4 with high predicted efficiency and low off-target effects. The Cas9 protein and guide RNAs (integrated DNA) were delivered to fertilised mouse zygotes as a ribonucleoprotein complex (Cas9 protein: 50 ng/µl, sgRNA: 2.5 ng/µl), and the edited zygotes were transferred into the uterine horns of pseudopregnant CFW/crl mice. The deletion and decreased expression of Ctla4 were confirmed by Sanger sequencing and flow cytometry. CTV-labeled splenic CD4^+^ T cells from *Ctla4*^*+/+*^ or *Ctla4*^*+/−*^ mice were stimulated with plate-coated anti-CD3 antibody (2 µg/ml; BD, 553058), B cells (4T: 1B), IL-21 (20 ng/ml; R&D Systems, 594-ML-010) and IL-2 (5 ng/ml; Miltenyi Biotec, 130-120-662) for 5 days. All mouse experiments and procedures were approved by the Australian National University Animal Experimentation Ethics Committee.

### Mouse VACV infection and anti-CTLA4 treatment

Vaccinia Western Reserve (VACV) was grown and titrated in BHK-1 cells maintained in DMEM with high glucose supplemented with 10% FBS and L-glutamine by The Tscharke Group at ANU. C57BL/6 female mice at 8–10 weeks of age were infected with 1 × 10^6^ plaque-forming units (pfu) of VACV in 200 μl PBS by the intraperitoneal route at Day 0, and 100 μg Armenian hamster anti-mouse CTLA4 antibody (UC10-4F10-11, BioXCell) or isotype control IgG (BioXCell) in 200 μl PBS was injected into mice at Days –1 and 3 by the intravenous route and at Day 5 by the intraperitoneal route. Mice were euthanised on Day 7, and the spleens were collected.

### Flow cytometry

Single-cell suspensions were prepared from human and mouse samples. Cells were stained with antibodies, and the dyes are listed in Supplementary Table 7. Antibody cocktails were prepared in cold flow cytometry buffer (PBS with 2% FBS).

Fixation was performed using Fix I buffer (BD, 557870) after cell surface marker staining. Before intracytoplasmic protein staining, a Cytofix/Cytoperm kit (BD, 554714) or 1% paraformaldehyde (Proscitech, C004) followed by 0.25% saponin (Sigma-Aldrich, 47036) was utilised for human and mouse samples, respectively. Intranuclear molecule staining was performed using Transcription Factor Buffer (BD Pharmingen, 562574) or a Foxp3/Transcription Factor Fixation/Permeabilisation kit (eBioscience, 00-5523-00) according to the manufacturers’ instructions.

For cytokine staining, 10^6^ PBMCs from *CTLA4*^+/−^ individuals and healthy donors were incubated with 2 μl of Leukocyte Activation Cocktail containing PMA, ionomycin and brefeldin A (BD, 550583) in 1 ml of culture medium for 6 h. After surface staining, cells were fixed/permeabilised (BD Cytofix/Cyotperm) and then stained with antibodies against IFN-γ and other cytokines. For CD107a degranulation analysis, PBMCs from *CTLA4*^+/−^ individuals and healthy donors were stimulated with PMA (25 ng/ml) and ionomycin (0.5 μg/ml) for 4 h, followed by staining with anti-LAMP1 antibodies (Sigma, L1418) and goat anti-rabbit IgG cross absorbed AF647 (Invitrogen, A-21245).

Flow cytometry data were collected with the FACS Canto, LSRII and Fortessa machines (BD, Biosciences) and analysed using FlowJo software v10 (FlowJo), and cell sorting was achieved with the FACS Aria II and Fusion machines (BD, Biosciences) at John Curtin School of Medical Research Imaging & Cytometry Facility with >95% single-cell population purity.

### Bulk RNA sequencing

Live CD57^+^ CD4^+^ T_EM_ (CD4^+^ CD8^−^ CD57^+^ CCR7^−^ CD45RA^−^), CD57^+^ CD4^+^ T_EMRA_ (CD4^+^ CD8^−^ CD57^+^ CCR7^−^ CD45RA^+^), CD57^−^ CD4^+^ T_EM_ (CD4^+^ CD8^−^ CD57^−^ CCR7^−^ CD45RA^−^) and CD57^+^ CD8^+^ (CD4^−^ CD8^+^ CD57^+^) cells were purified from fresh PBMCs of three healthy blood donors by flow cytometry. Total RNA was then immediately extracted by a GenElute Total RNA isolation kit (Sigma, RNB100) with genomic DNA digestion (Sigma, DNASE10) according to the manufacturer’s instructions, and the quality and integrity of RNA samples were validated by a Bioanalyzer (Agilent) with RIN values >8.0. The mRNA library was prepared by the Biomolecular Resource Facility at ANU using the TruSeq Stranded mRNA Library Prep kit (Illumina, 20020594) and sequenced in one high-output flow cell with 75 bp single-end reads by a NextSeq500 sequencer (Illumina). Each sample had coverage of at least 25 million reads.

### Bulk RNA sequencing data analysis

The RNA sequencing data were submitted to The ANU Bioinformatics Consultancy for downstream analysis. Generally, all 12 samples were evaluated to be of good quality by FastQC; the reads were aligned to the human genome GRCh38 (hg38) by HISAT2 (v2.1.0) and sorted with SAMtools [84, 85], and mapped reads were assigned and counted based on NCBI Refseq gene annotations by FeatureCount (v1.4) [86]. After filtering out genes expressed at low levels across all the samples, the remaining genes with at least 0.5 counts per million reads in more than three samples were included for downstream analyses. Gene read libraries were normalised by the trimmed mean of M values (TMM) method [87], and data were transformed by voom with sample quality adjustment [88]. Then, the data were fitted in a linear model with moderated t-statistics with the empirical Bayes method [89], and differentially expressed genes with a Benjamini–Hochberg adjusted *P* value <0.05 were identified and visualised with Glimma [90]. GSEA was performed by the CAMERA method accounting for intergene correlation based on the MSigDB [91]. All statistical analyses were performed by the limma package [89].

### Single-cell RNA-seq for gene expression and VDJ profiling

CD57^+^ CD4^+^ T cells (7AAD^−^ CD19^−^ CD3^+^ CD4^+^ CD8^−^ CD57^+^) and CD57^−^ CD4^+^ T cells (7AAD^−^ CD19^−^ CD3^+^ CD4^+^ CD8^−^ CD57^−^) from tonsil and blood samples from one child were purified into 1.5-ml Eppendorf tubes with flow cytometry buffer. Single-cell gene expression and VDJ libraries were prepared by the Biomolecular Resource Facility at ANU using the Chromium Next GEM Single Cell 5’ Kit v2 Dual Index and Single Cell Human TCR Amplification Kit (10× Genomics) according to the manufacturer’s instructions. The library was sequenced in an S1 flow cell with 2 × 50 bp paired-end reads by a NovaSeq 6000 sequencer (Illumina). The 10X Cell Ranger package (10X Genomics, v6.0.1) was used to process data and align to the human genome (GRCh38-2020-A).

### Single-cell RNA-seq analysis

The Seurat package (v4.1.0) was utilised for gene expression analyses with a standard processing workflow. Quality control was performed to filter out low-quality cells (doublets and dead cells). Cells with between 200 and 2500 detected genes and a percentage of mitochondrial reads less than 5% were selected for downstream analyses. Unwanted CD19^+^ B cells, CD8^+^ T cells, and CD14^+^ and CD300E^+^ monocytes (approximately 1% in total) were removed from the datasets. Highly variable TRBV and TRAV genes were excluded from downstream gene expression and cell cluster analyses. UMI count data were normalised by regularised negative binomial regression (SCTransform) [92]. Dimensionality reduction, cell clustering and differentially expressed feature analyses were performed with the default settings. Samples were then integrated using anchors identified with the FindIntegrationAnchors function [93], and their similarities and differences were compared after normalisation by SCTransform. According to the ElbowPlot results, 30, 30 and 40 principal components were utilized for UMAP analyses for integrated CD57^+^ T cells, tonsillar cells and blood cells, respectively. The FindClusters function was then run to identify clusters using resolutions of 0.5, 0.6, and 0.62 to separate the classic T-cell subsets. The cluster biomarkers in each subset compared to all other cells were identified with the FindAllMarkers function, and partial feature genes were selected from the top 200 DEGs for heatmap analyses.

The ScRepertoire package (v1.3.5) was used to analyze TCR clonotypes [94]. The VDJ contigs assembled using the Cell Ranger pipeline were extracted from each sample and then combined. Clonal space homeostasis examining the relative space occupied by clones at specific proportions was performed for tonsillar and blood CD57^+^ and CD57^−^ CD4^+^ T cells. The similarity measurements between the clonotypes from different samples were determined using the overlap coefficient method. The visualization for the direct comparison of clonotypes between the two samples was performed by the scatterClonotype function.

All analyses were performed in R using RStudio (v.1.4.1717). The materials used for the analysis are listed in Supplementary Table 7.

### CTLA4 recycling and intracellular expression assessment for *CTLA4*^+/−^ individuals

PBMCs from *CTLA4*^+/−^ individuals or healthy donors were stimulated with anti-CD3/28 beads (1 bead: 2 cells, Miltenyi Biotec, 130-091-441) in RPMI-1640 with L-glutamine (Thermo Fisher) supplemented with 10% FBS (Thermo Fisher), 100 U/ml penicillin and streptomycin (Sigma-Aldrich) and 100 mM HEPES (Thermo Fisher) for 6 or 24 h. Cells were incubated with anti-CTLA4 antibody (BD, 555853) during the incubation to stain recycling/trafficking CTLA4 or fixed and permeabilised after the incubation for intracellular CTLA4 staining (BioLegend, 349914).

### CD80/86 transendocytosis in CTLA4-expressing cells

To compare CD80/86 transendocytosis via CTLA4 in different CD4^+^ T cells, live naïve cells (CD3^+^ CD4^+^ CD25^−^ CXCR5^−^ PD-1^−^ CD45RA^+^), GC-Tfh (CD3^+^ CD4^+^ CD25^−^ CXCR5^hi^ PD-1^hi^ CD45RA^−^), Tregs (CD3^+^ CD4^+^ CD25^+^ CXCR5^int/low^ PD-1^int/low^ CD45RA^−^) and Tfr (CD3^+^ CD4^+^ CD25^+^ CXCR5^hi^ PD-1^hi^ CD45RA^−^) were purified from human tonsils by flow cytometry. Irradiated Raji cells (50 Gy) were stained with CTV (Thermo Fisher) for identification and then incubated with different purified CD4^+^ T-cell subsets (20 T: 1 Raji) for 1, 2, 6, 12 and 24 h. After incubation, the CD80/86 levels in live CTV^+^ CD19^+^ CD4^−^ irradiated Raji cells were assessed by flow cytometry.

To investigate the effect of CTLA4 point mutation, the *CTLA4* c.412C>G mutation was introduced into a human CTLA4 cDNA expression plasmid (C-GFPSpark tagged, Sino Biological; HG11159-ACG) by a Q5 site-directed mutagenesis kit (NEB, E0554) with specific primers (F - ATGAGCTCCACCTTGCAG; R - GTACCCACCGgCATACTACCT) and validated by Sanger sequencing. A plasmid with the *CTLA4* c.208C>T mutation was also prepared as a positive control with different primers (F - CACTGAGGTCtGGGTGACAGT; R - GCTTTGCCTGGAGATGCATA). Five micrograms of WT or mutant CTLA4 plasmids or empty vector (pCMV3-C-GFPSpark; Sino Biological; CV026) were transfected into 800,000 Jurkat cells by the Neon system (1350 V, 10 ms pulse width, 3 pulses; Invitrogen). After overnight incubation, CTLA4-transfected Jurkat cells were incubated with CTV-labeled irradiated Raji cells (20 Jurkat: 1 Raji) for 24 h, and then the CD80/86 levels of live CTV^+^ CD19^+^ CD4^−^ irradiated Raji cells were assessed by flow cytometry.

### CD57^+^ CD4^+^ T-cell cytotoxicity against Raji cells

Live blood CD57^+^ CD4^+^ T cells (7AAD^−^ CD19^−^ CD3^+^ CD8^−^ CD57^+^) and CD57^+^ CD4^+^ T cells (7AAD^−^ CD19^−^ CD3^+^ CD8^−^ CD57^−^) were purified from fresh PBMCs of healthy donors by flow cytometry. Raji cells were pulsed with SEB (Sigma-Aldrich, S4881), and 1.5 × 10^6^ Raji cells were incubated with 1.9 μg SEB in 100 µl culture medium for 15 min [60]. After cell washing, 10,000 SEB-pulsed Raji cells were cocultured with blood CD57^+^ or CD57^−^ CD4^+^ T cells at different ratios (ranging from 1 T:1 R to 20 T:1 R). After brief centrifugation (400 g, 2 min), the cells were incubated for 24 h, followed by Annexin V and 7AAD analysis.

To detect granzyme B cleavage activity in Raji cells incubated with blood CD57^+^ CD4^+^ T cells, a granzyme B specific, cell-permeable and fluorogenic substrate was utilised from the PanToxiLux kit (OncoImmunin, PTL802-8). Live blood CD57^+^ and CD57^−^ CD4^+^ T cells were purified from healthy donors by flow cytometry, and Raji cells were pulsed with SEB as described above. A total of 10,000 SEB-pulsed Raji cells labeled with *TFL4* dye were incubated with CD57^+^ CD4^+^ or CD57^−^ CD4^+^ cells (10 T:1 Raji). After removing the culture medium, cells were resuspended in 75 μl of the granzyme B-specific substrate *PS*. After brief centrifugation (400 g, 2 min), the cells were incubated for 2 h. The granzyme B-specific *PS* fluorescence signal in TLF4^+^ Raji cells was assessed by flow cytometry.

### CD57^+^ CD4^+^ T-cell cytotoxicity against primary B cells

Live blood CD57^+^ CD4^+^ T cells (7AAD^−^ CD19^−^ CD3^+^ CD8^−^ CD57^+^), CD57^+^ CD4^+^ T cells (7AAD^−^ CD19^−^ CD3^+^ CD8^−^ CD57^−^) and B cells (7AAD^−^ CD19^+^ CD3^−^) were purified from fresh PBMCs of healthy donors by flow cytometry. B cells were incubated with CD57^+^ or CD57^−^ CD4^+^ T cells for 24 h (10 T: 1 B) in the presence or absence of SEB (200 ng/ml) with recombinant human IL-4 (20 ng/ml; Miltenyi Biotec, 130-093-919) and IL-21 (20 ng/ml; Miltenyi Biotec, 130-095-784), followed by Annexin V and 7AAD analysis.

### Plasmablast development

Live CD4^−^ CD19^+^ B cells and CD19^−^ CD57^+^ CD4^+^ and CD19^−^ CD57^−^ CD4^+^ T cells were purified from fresh PBMCs of healthy donors, and B cells were stained with CTV and incubated with CD57^+^ CD4^+^ or CD57^−^ CD4^+^ T cells for 6 days (1 T:1 B) in the presence of SEB (200 ng/ml) or anti-CD2/3/28 beads (1 bead:2 T) with recombinant human IL-4 (20 ng/ml) and IL-21 (20 ng/ml), and 7AAD^−^ CD4^−^ CD19^+^ CTV^+^ CD27^hi^ CD38^hi^ plasmablasts were assessed by flow cytometry.

### Suppression of increased granzyme and decreased TCF1 expression by soluble CTLA4-Ig

PD-1^−^ CD4^+^ T cells were purified from human tonsils, labeled with CTV, and incubated with anti-CD3 beads (1 bead:2 cells) and CD80/86-expressing irradiated Raji cells (2 T:1 Raji) with recombinant human IL-2 (50 ng/ml; Thermo Fisher, RIL2I) or IL-21 (50 ng/ml, Miltenyi Biotec) with or without 1.5 μg/ml soluble CTLA4-Ig (Chimerigen, CHI-HF-210A4-C100), and granzyme A and TCF1 expression was assessed at Day 4.

### Statistical analysis

Two-tailed unpaired *t*-tests, one-way ANOVA and two-way ANOVA with Bonferroni’s multiple comparison test were performed according to the experimental designs. A paired *t*-test was only performed for the analysis of PBMCs from cancer patients before and after checkpoint therapy. A two-tailed Pearson correlation coefficient was calculated to reveal the relationship between the CD57^+^ CD4^+^ cell proportion and blood memory B-cell count in patients with PAD. All statistical analyses were performed by GraphPad Prism 9 software.

## Supplementary information

Supplementary table 1

Supplementary table 2

Supplementary table 3

Supplementary table 4

Supplementary table 5

Supplementary table 6

Supplementary table 7

Supplementary figures

Supplementary figure legends

## Data Availability

RNA-seq data were deposited in Gene Expression Omnibus (GEO) under GSE171082. Single-cell RNA-seq data with gene expression and VDJ profiling data were deposited in GEO under GSE200257. Data are available as of the date of publication.
