# Supplementary material for: CTLA4 protects against maladaptive cytotoxicity during the differentiation of effector and follicular CD4+ T cells
Source: Cell Mol Immunol. 2023 May 9;20(7):777–93. doi: 10.1038/s41423-023-01027-8 (PMC10166697; doi:10.1038/s41423-023-01027-8)

Supplementary Fig 1

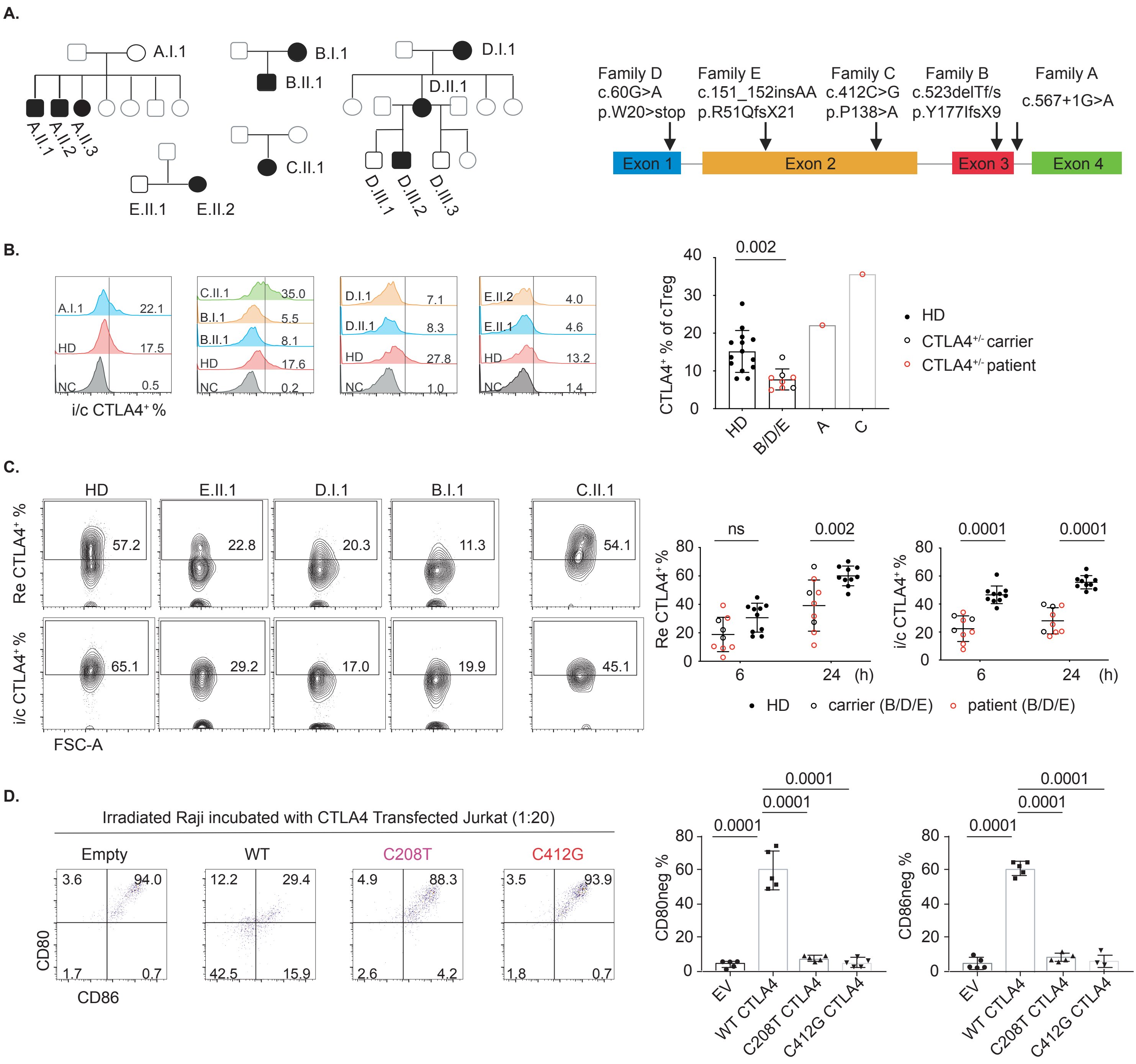

Supplementary Fig 2

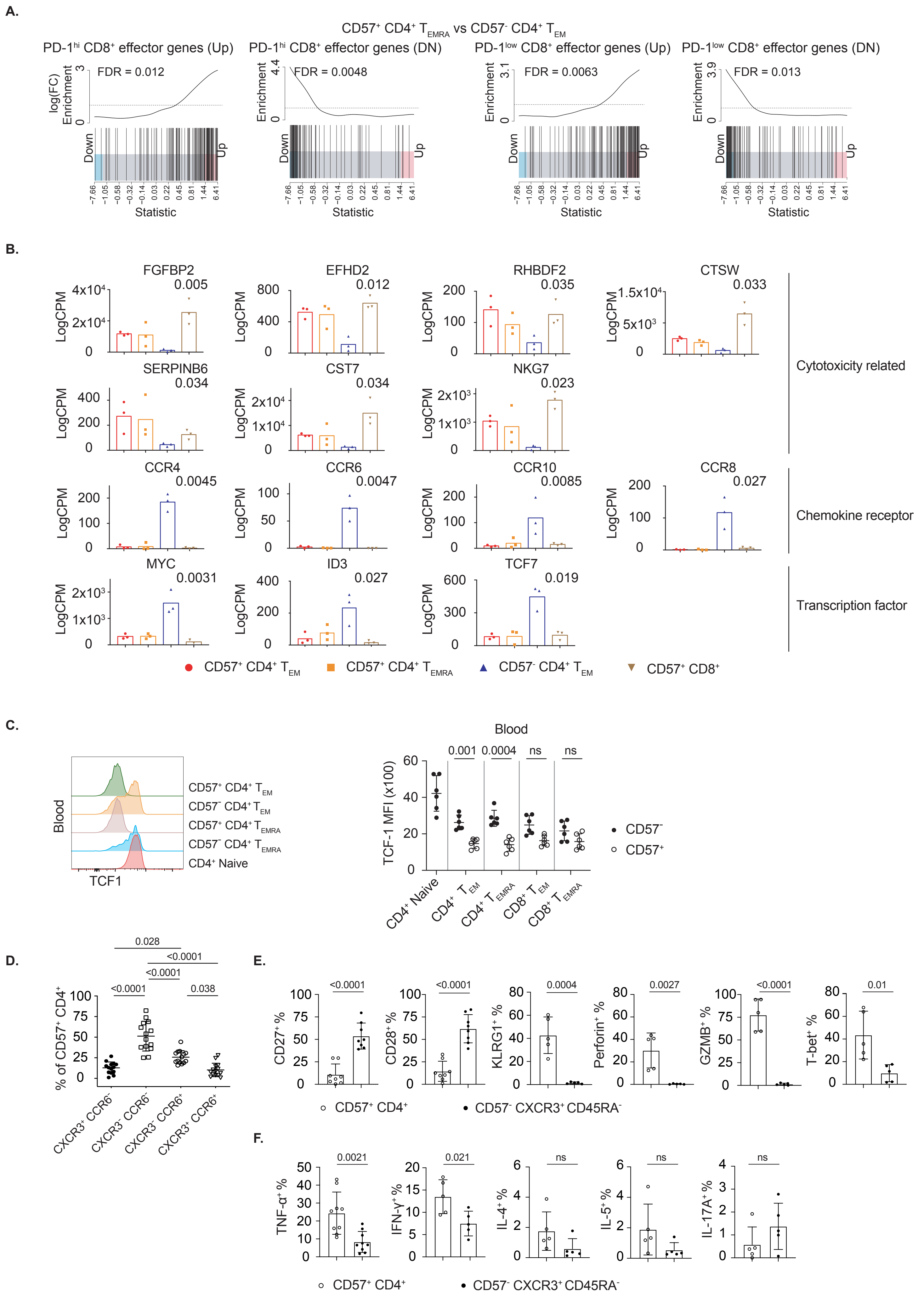

Supplementary Fig 3

A.

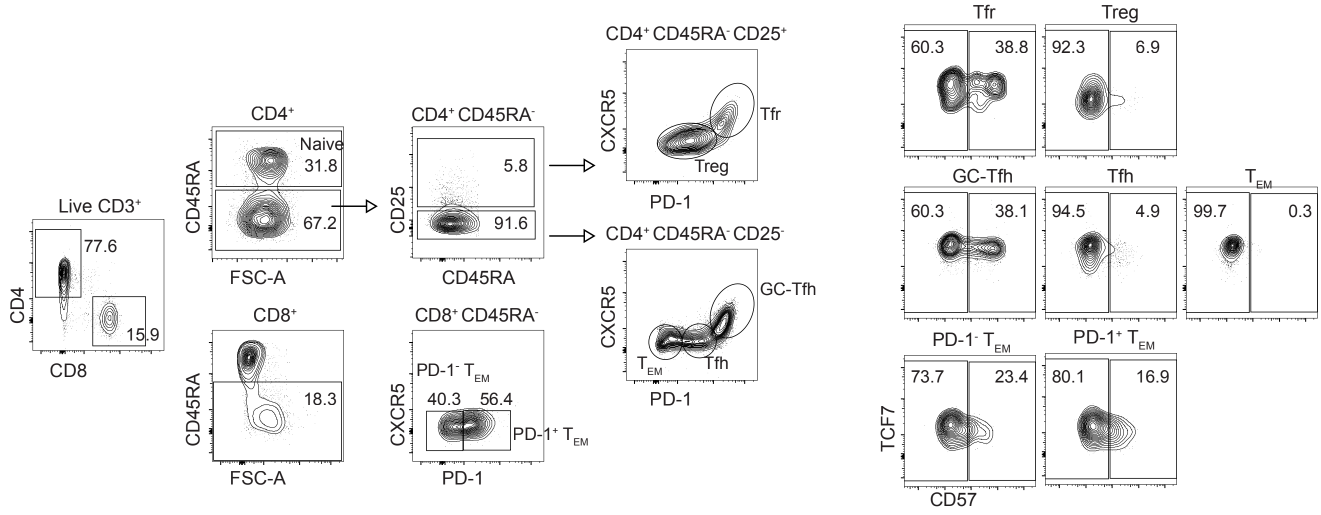

B.

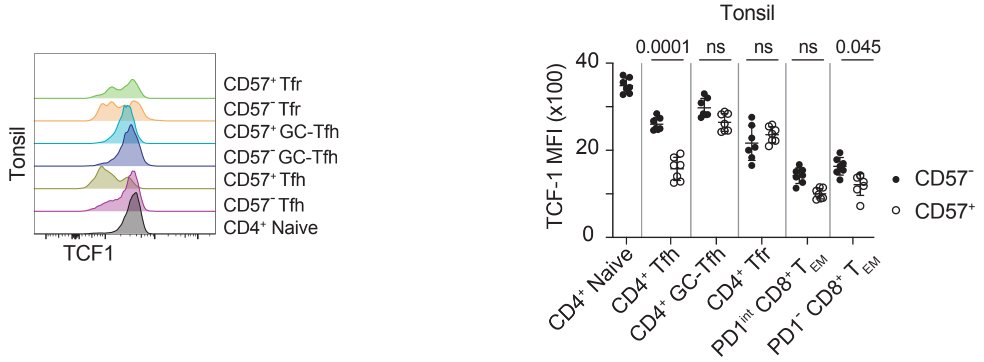

Supplementary Fig 4

A.

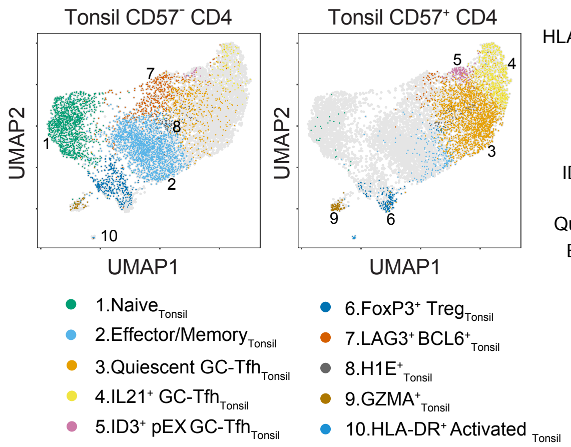

B.

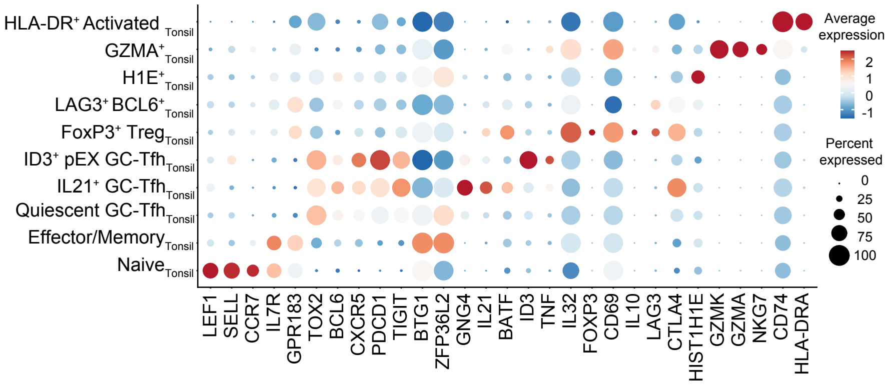

C.

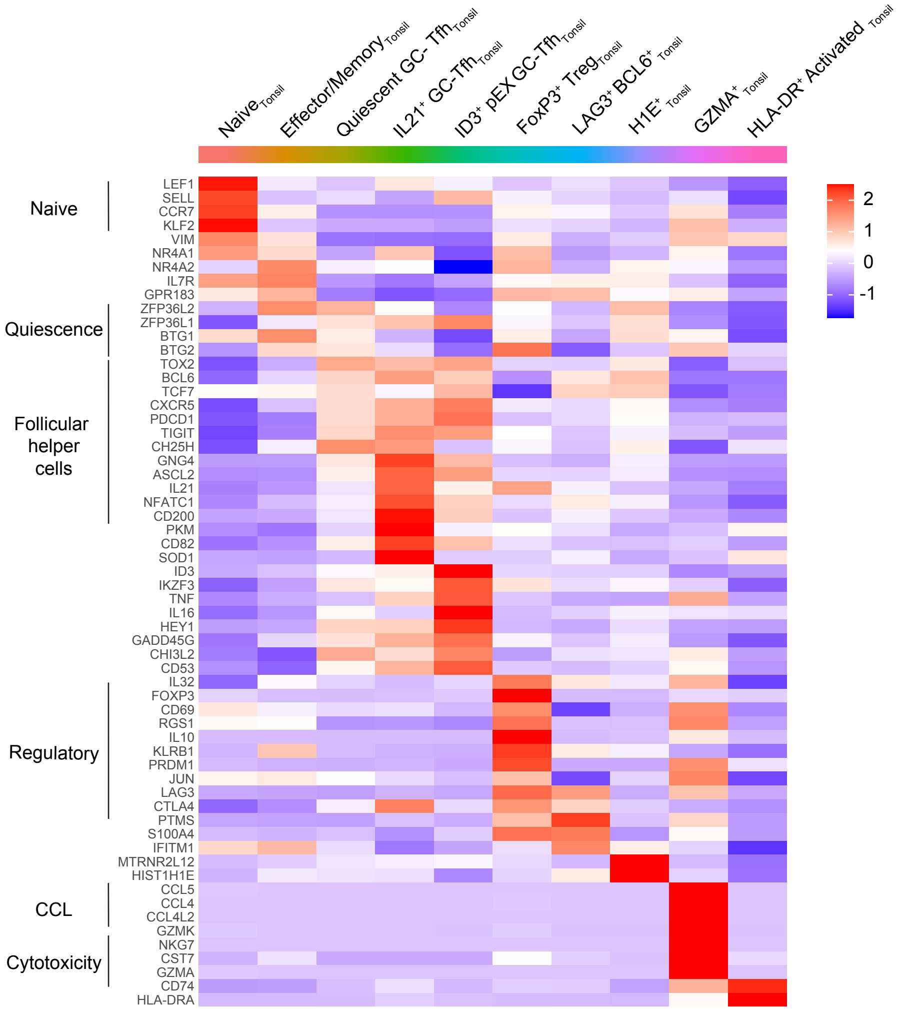

Supplementary Fig 5

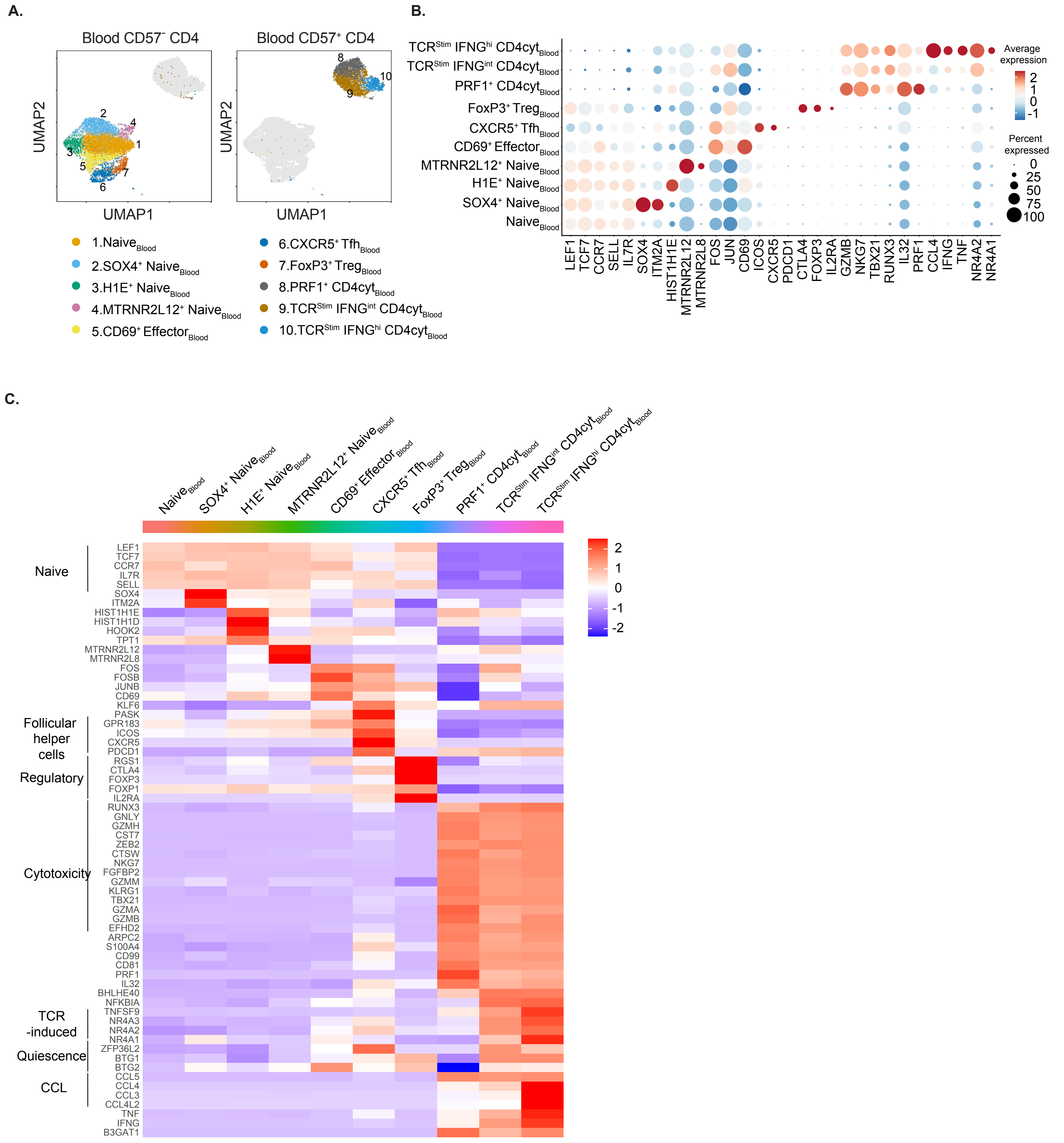

Supplementary Fig 6

A.

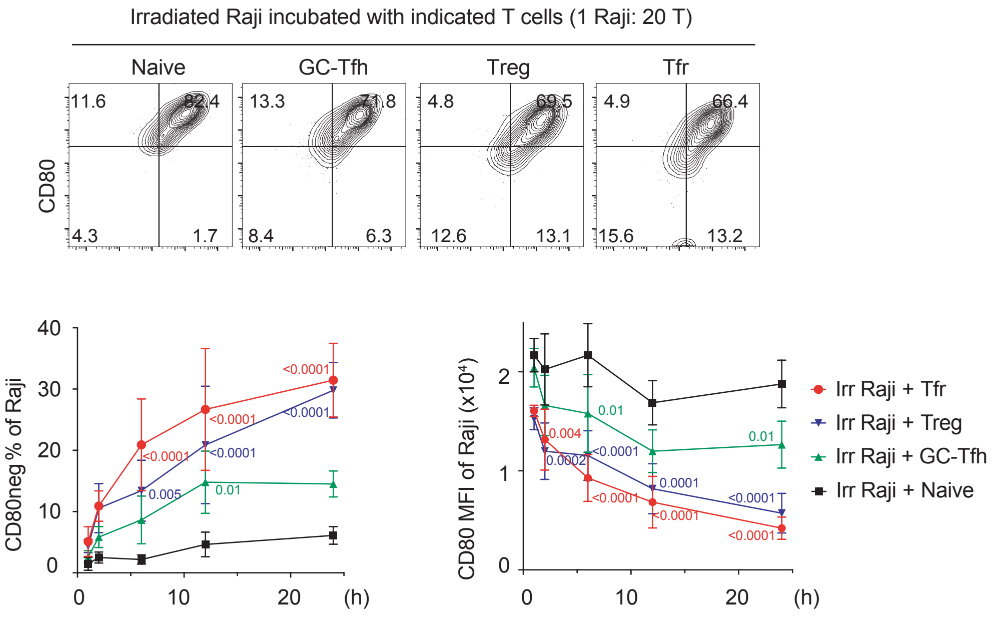

Supplementary Fig 7

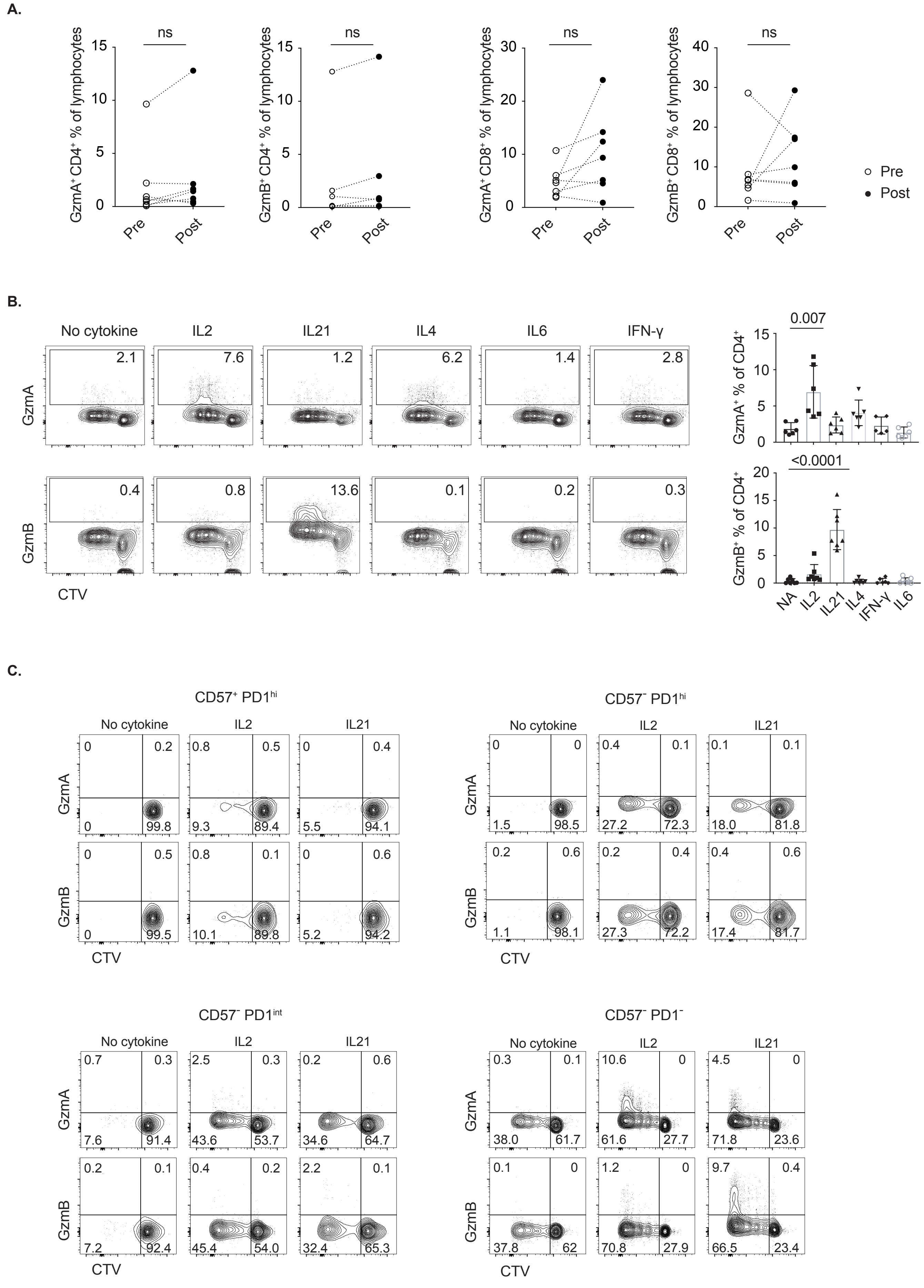

Supplementary Fig 8

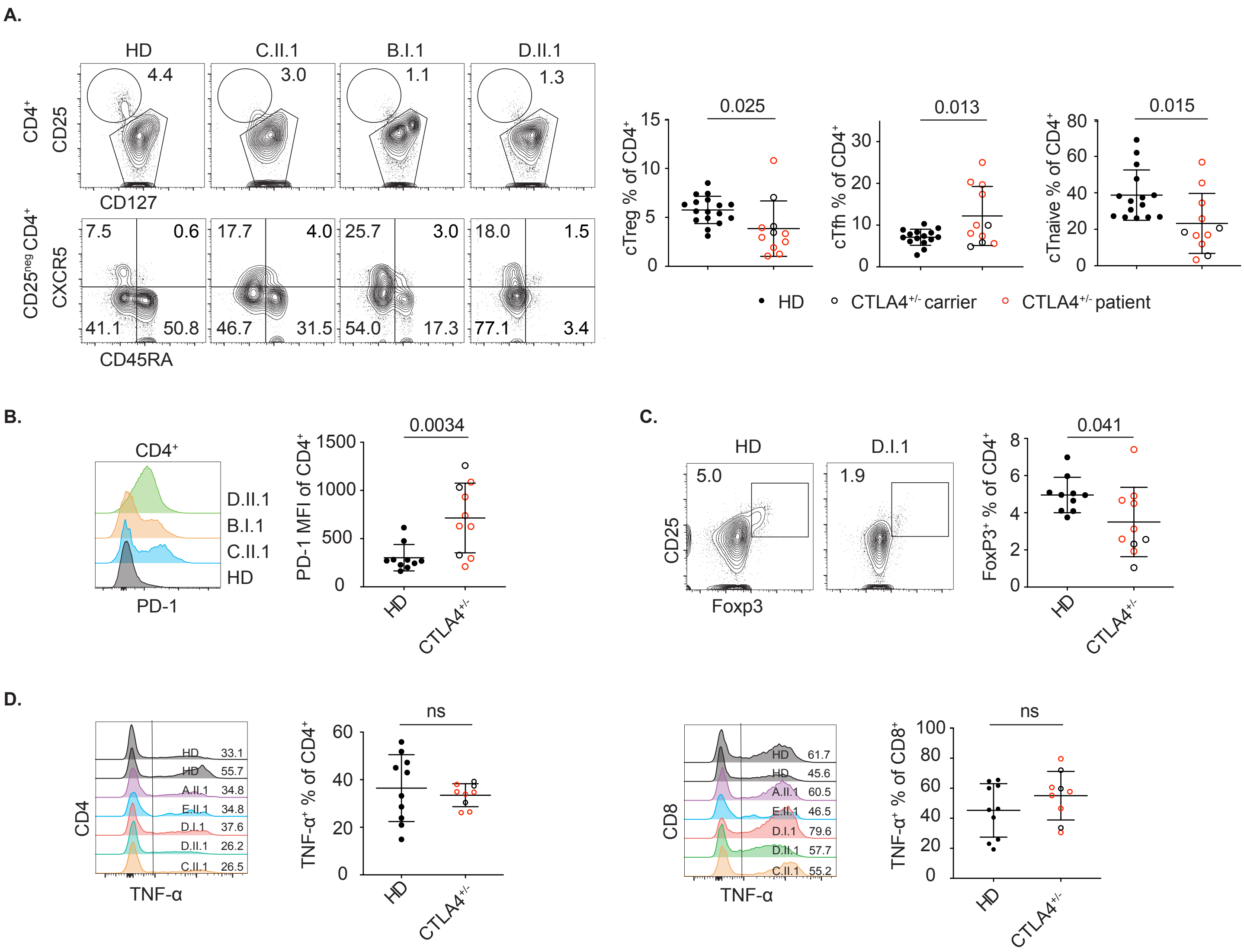

Supplement: Supplementary file 8 — Supplementary figures [file 41423_2023_1027_MOESM8_ESM.pdf]
