# Supplementary material for: CTLA4 protects against maladaptive cytotoxicity during the differentiation of effector and follicular CD4+ T cells
Source: Cell Mol Immunol. 2023 May 9;20(7):777–93. doi: 10.1038/s41423-023-01027-8 (PMC10166697; doi:10.1038/s41423-023-01027-8)
Supplement: Supplementary file 9 — Supplementary figure legends [file 41423_2023_1027_MOESM9_ESM.docx]

**Supplementary figure titles and legends**

**Supplementary Fig. 1** Impaired CTLA4 expression and function is conferred by human *CTLA4* mutations.

**A** *CTLA4*^+/-^ pedigrees with *CTLA4* genotypes indicated. Gray symbols, WT; black empty symbols, unaffected carriers of *CTLA4* mutations; black solid symbols, affected patients with *CTLA4* mutations. **B** Intracellular CTLA4 expression in resting CD25^+^ CD127^low^ CD4^+^ blood Tregs from PBMCs of healthy donors (n = 14; black solid symbols) and *CTLA4*^+/-^ individuals (n = 10; carriers - black empty symbols, patients - red empty symbols). Premature stop codons (c.523delTf/s; c.60G>A; c.151_152insAA) reduced CTLA4 levels, while the point mutation c.412C>G did not alter CTLA4 expression. **C** Representative and summary data of CTLA4 recycling and intracellular CTLA4 expression in blood CD25^+^ CD127^low^ CD4^+^ cells from PBMCs of healthy donors (n = 10) and *CTLA4*^+/-^ individuals treated in the presence or absence of anti-CD3/28 beads for 6 and 24 hours. **D** Representative flow micrographs and quantification of irradiated Raji cell CD80/86 transendocytosis by Jurkat cells transfected with WT or mutant *CTLA4* (n = 5). CTV-labeled irradiated Raji cells were incubated with Jurkat cells carrying WT or mutant C412G *CTLA4* (1 Raji:20 Jurkat) for 24 hours. Cells with the *CTLA4*^C208T^ point mutation, which is known to confer impaired CD80/86 binding, served as the positive control. Summary data (mean ± SD) were collected from 2-3 independent experiments, and statistical analyses were performed by two-tailed unpaired *t* tests (**B**) and one-way (**D**) or two-way (**C**) ANOVA with Bonferroni's multiple comparison tests. P values are shown. NS, not significant.

**Supplementary Fig. 2** Blood CD57^+^ CD4^+^ T cells have a cytotoxic transcriptome marked by TCF1 downregulation.

**A** Gene set enrichment analysis plots for the indicated gene sets (CCR7^-^ PD-1^hi^ or CCR7^-^ PD-1^low^ CD8^+^ vs. CCR7^+^ CD45RA^+^ CD8^+^) in the comparison of transcriptomes between CD57^+^ CD4^+^ T_EMRA_ and CD57^-^ CD4^+^ T_EM_. **B** Select transcripts of differentially expressed genes between CD57^+^ CD4^+^ T_EM_ and CD57^-^ CD4^+^ T_EM_ in all four indicated cell subsets (log counts per million with the FDR value indicated). **C** TCF1 expression in different CD4^+^ T-cell subsets from PBMCs of healthy donors (n = 6). **D** CXCR3 and CCR6 expression in CD57^+^ CD4^+^ T cells from PBMCs of healthy donors (n = 14). **E** CD27, CD28, KLRG1, perforin, GzmB and T-bet expression in CD57^+^ CD4^+^ T cells from PBMCs of healthy donors (n = 5-8). **F** Expression of the cytokines TNF-$\alpha$, IFN-γ, IL-4, IL-5 and IL-17A in CD57^+^ CD4^+^ T cells from PBMCs of healthy donors (n = 5-9). Summary data (mean ± SD) were collected from 2-3 independent experiments, and statistical analyses were performed by two-tailed unpaired *t* tests (**E**, **F**) and one-way ANOVA with Bonferroni's multiple comparison tests (**C**, **D**). P values are shown. NS, not significant.

**Supplementary Fig. 3** TCF1 expression in tonsillar CD57^+^ CD4^+^ T-cell subsets.

**A** Flow cytometry gating strategy for CD4^+^ Tfr, CD4^+^ Tregs, CD4^+^ GC-Tfh, CD4^+^ Tfh, CD4^+^ T_EM_, CD4^+^ naive cells, CD8^+^ PD-1^-^ T_EM_ and CD8^+^ PD-1^+^ T_EM_ from tonsil samples. **B** TCF1 expression in different CD4^+^ T-cell subsets in tonsils from donors who underwent tonsillectomy (n = 6). Summary data (mean ± SD) were collected from 2-3 independent experiments, and statistical analyses were performed by one-way ANOVA with Bonferroni's multiple comparison tests (**B**). P values are shown. NS, not significant.

**Supplementary Fig. 4** Single-cell RNA-seq analysis of tonsillar CD57^-^ and CD57^+^ CD4^+^ T cells.

**A** Uniform manifold approximation and projection (UMAP) plot of the single-cell RNA-seq data from 3597 purified tonsillar CD57^+^ CD4^+^ T cells and 5056 purified tonsillar CD57^-^ CD4^+^ T cells. **B** Dot plot showing the canonical markers of differentially expressed genes for tonsillar CD4^+^ T-cell subsets. **C** Heatmap showing the relative expression of select genes among the top 200 differentially expressed genes in every tonsillar CD4^+^ T-cell subset compared to all other subsets.

**Supplementary Fig. 5** Single-cell RNA-seq analysis of blood CD57^-^ and CD57^+^ CD4^+^ T cells.

**A** Uniform manifold approximation and projection (UMAP) plot of the single-cell RNA-seq data from 3265 purified blood CD57^+^ CD4^+^ T cells and 5953 purified blood CD57^-^ CD4^+^ T cells. **B** Dot plot showing the classical markers of differentially expressed genes for blood CD4^+^ T-cell subsets. **C** Heatmap showing the relative expression of select genes among the top 200 differentially expressed genes in every blood CD4^+^ T-cell subset compared to all other subsets.

**Supplementary Fig. 6** CD80 transendocytosis by different tonsillar CD4^+^ T-cell subsets.

**A** Representative and summary data of CD80 transendocytosis by CTLA4-expressing tonsillar CD4^+^ T cells. CTV-labeled irradiated Raji cells were incubated with tonsillar Tfr, Tregs, GC-Tfh and naive CD4^+^ T cells (n = 4-5) for 1, 2, 6, 12 and 24 hours (1 Raji:20 T cells). Summary data (mean ± SD) were collected from 3 independent experiments, and statistical analyses were performed by two-way ANOVA with Bonferroni's multiple comparison tests. P values are shown. NS, not significant.

**Supplementary Fig. 7** Granzyme expression induction by CD4^+^ T cells in response to stimulation.

**A** Proportions of GzmA^+^ and GzmB^+^ CD4^+^ and CD8^+^ T cells from PBMCs of cancer patients (n = 7) after treatment with ipilimumab/nivolumab (anti-CTLA4/PD-1). Each symbol represents one cancer patient before (*empty symbols*) and after (*filled symbols*) treatment. **B** Induced GzmA and GzmB expression by CD4^+^ T_EM_ (n = 6) in response to different cytokines. Tonsillar CD4^+^ T_EM_ (CD57^-^ PD-1^-^) were stimulated with anti-CD3 beads and CD80/86-expressing irradiated Raji cells (2 T:1 Raji) with IL-2 (50 ng/ml), IL-21 (50 ng/ml), IL-4 (20 ng/ml), IL6 (10 ng/ml) or IFN-γ (10 ng/ml) for 4 days. **C** Representative flow cytometry data of cell proliferation and granzyme expression for tonsillar CD4^+^ T-cell subsets upon stimulation. Tonsillar CD57^-^ PD-1^-^ CD4^+^, CD57^-^ PD-1^int^ CD4^+^, CD57^-^ PD-1^hi^ CD4^+^ and CD57^+^ PD-1^hi^ CD4^+^ T cells were stimulated with IL-2 or IL-21 as described above. Representative and summary (mean ± SD) data were collected from 2-3 independent experiments, and statistical analyses were performed by two-tailed paired *t* tests (**A**) and one-way ANOVA with Bonferroni's multiple comparison tests (**B**). P values are shown. NS, not significant.

**Supplementary Fig. 8** Increased CD4^+^ T-cell activation is conferred by human *CTLA4* deficiency.

**A** Flow cytometric analysis of blood Tregs, Tfh and naïve CD4^+^ T cells from PBMCs of healthy donors (n = 17) and *CTLA4*^+/-^ individuals (n = 11; carriers - black empty symbols, patients - red empty symbols). **B** Flow cytometric analysis of PD-1 expression by blood CD4^+^ T cells from PBMCs of healthy donors (n = 10) and *CTLA4*^+/-^ individuals (n = 10). **C** Flow cytometric analysis of Foxp3 expression by CD4^+^ T cells from PBMCs of healthy donors (n = 10) and *CTLA4*^+/-^ individuals (n = 10). **D** Flow cytometric analysis of TNF-$\alpha$ expression by CD4^+^ and CD8^+^ T cells from PBMCs of healthy donors (n = 10) and *CTLA4*^+/-^ individuals (n = 9). Summary data (mean ± SD) were collected from 2-3 independent experiments, and statistical analyses were performed by two-tailed unpaired *t* tests. P values are shown. NS, not significant.
